# Expression patterns of Slit and Robo family members in adult mouse spinal cord and peripheral nervous system

**DOI:** 10.1371/journal.pone.0172736

**Published:** 2017-02-24

**Authors:** Lauren Carr, David B. Parkinson, Xin-peng Dun

**Affiliations:** 1 Plymouth University Peninsula Schools of Medicine and Dentistry, Plymouth, Devon, United Kingdom; 2 Hubei University of Science and Technology, Xian-Ning City, Hubei, China; University of Edinburgh, UNITED KINGDOM

## Abstract

The secreted glycoproteins, Slit1-3, are classic axon guidance molecules that act as repulsive cues through their well characterised receptors Robo1-2 to allow precise axon pathfinding and neuronal migration. The expression patterns of Slit1-3 and Robo1-2 have been most characterized in the rodent developing nervous system and the adult brain, but little is known about their expression patterns in the adult rodent peripheral nervous system. Here, we report a detailed expression analysis of Slit1-3 and Robo1-2 in the adult mouse sciatic nerve as well as their expression in the nerve cell bodies within the ventral spinal cord (motor neurons) and dorsal root ganglion (sensory neurons). Our results show that, in the adult mouse peripheral nervous system, Slit1-3 and Robo1-2 are expressed in the cell bodies and axons of both motor and sensory neurons. While Slit1 and Robo2 are only expressed in peripheral axons and their cell bodies, Slit2, Slit3 and Robo1 are also expressed in satellite cells of the dorsal root ganglion, Schwann cells and fibroblasts of peripheral nerves. In addition to these expression patterns, we also demonstrate the expression of Robo1 in blood vessels of the peripheral nerves. Our work gives important new data on the expression patterns of Slit and Robo family members within the peripheral nervous system that may relate both to nerve homeostasis and the reaction of the peripheral nerves to injury.

## Introduction

The Slit axon guidance molecules and their receptors, known as Robo (Roundabout), form one of the most crucial ligand-receptor pairings among the classic axon guidance signaling pathways by serving as a repellent to allow precise axon pathfinding and neuronal migration during development [1–10]. So far, three Slit ligands (Slit1-3) have been identified in vertebrates with a spatio-temporal expression pattern in the nervous system as well as in the peripheral tissue and other organs during development [11–14]. Using Slit or Robo gene null fruit flies or mice as research models, Slit-Robo interactions have been shown to act as a repulsive signal to regulate actin dynamics for axon guidance at the midline for commissural, retinal, olfactory, cortical and precerebellar axons [1–10]. Validated by numerous studies, the repulsive function of Slit1-3 though their Robo receptors is conserved in worms, flies and vertebrates [1–10]. In addition to these functions, more recent studies have also shown that they are important regulators for cell migration and angiogenesis during development [15–16].

Based on their homology, four Robo receptors (Robo1-4) have been identified in mammals [1–4, 17–18]. Robo1 and Robo2 are highly expressed in the nervous system during embryonic development and play a key role in axon guidance and cell migration in the developing nervous system [19]. Robo1 and Robo2 also show a tissue and organ specific expression pattern outside of the nervous system and their expression is required to regulate morphogenesis, for example, in the kidney, the lung and the heart [2, 11–14, 20]. In contrast, Robo3 is expressed only by commissural neurons and it plays a crucial role in the control of commissural axons crossing the midline of the central nervous system [17, 21–24]. Robo4 was first identified in 2002 and is a much smaller protein compared to Robo1-3 [18]. Robo4 possesses only two of the five immunoglobulin (Ig) domains and two of the three fibronectin domains present in the extracellular component of Robo1-3 [18]. Intensive studies have been carried out on Robo4 since its discovery and these studies have confirmed that Robo4 is an endothelial cell specific protein required for maintaining blood vessel integrity [25–30].

Although four Robo receptors have been identified in mammals, to date, Slit1-3 have been shown to bind only to the Robo1 and Robo2 receptors with high affinity in mammals [1–10]. Recently, Evans et al have reported that Drosophila midline glia express Robo2 and that Robo2 acts in trans to inhibit Slit-Robo1 repulsion and promote midline commissural axon crossing. This effect is mediated by Robo2-Robo1 interaction between their extracellular Ig1 and Ig2 domains [31]. The key amino acid residues required for Slit1-3 binding in Robo1 and Robo2 receptors have been identified by studying the crystal structure of human Slit2 and Robo1 interacting domains and consist of the second leucine rich domain in Slit1-3 and the immunoglobulin domain 1 (Ig1) in Robo1-2 [32]. Studies have revealed that mammalian Robo3 does not bind Slit1-3 with high affinity [17, 21–24]. In support of this finding, a recent study has shown that a few key residues required for Slit1-3 binding in the Ig1 domain of Robo1 and Robo2 have been substituted in the mammalian Robo3 receptor [33]. Instead of binding to Slit1-3, these changed residues now favour mammalian Robo3 forming a complex with the deleted in colorectal cancer (DCC) protein, a Netrin-1 receptor, to transduce Netrin-1 mediated attractive signaling [33]. It has been found that a conserved tyrosine residue in the intracellular domain of Robo3 could be phosphorylated upon Netrin-1 binding to its receptor DCC, providing more evidence for a functional interaction between the DCC and Robo3 receptors [33]. This conserved tyrosine residue phosphorylation in Robo3 contributes to the attractive actions of Netrin-1 thought its DCC receptor [33]. As for Robo3, there is little evidence for Robo4 acting as a receptor for Slit-dependent signaling [25–30]. In searching for Robo4 binding proteins, it has been found that Robo4 binds Unc5B, another Netrin-1 receptor expressed in endothelial cells [34]. The interaction between Robo4 and Unc5B on adjacent endothelial cells has been shown to be required for the maintenance of adult blood vessel integrity [34]. Robo4 is also expressed in hematopoietic stem cell where it cooperates with the Cxcr4 receptor to localize hematopoietic stem cells to bone marrow niches [35–36].

Slit1-3 have been well characterised as a potent repellent for axon pathfinding by binding to Robo1 and Robo2 receptors present on the growth cone [1–10]. However, recent studies have revealed that Slit or Robo could interact with other guidance molecules or receptors to initiate attractive signaling [37–38]. During development, the Drosophila embryonic contractile tissue is established by the migration and adhesion of muscles towards their corresponding tendon cells. In an effort to identify a tendon-specific signal that is essential for the guidance of migrating muscles towards tendon cells, Wayburn and Volk identified a novel tendon-specific transmembrane protein, which was named LRT due to the presence of a leucine-rich repeat domain in its extracellular region. LRT forms a protein complex with Robo and the LRT-Robo interaction initiates an attractive signaling for muscle migration towards the tendon cells [37]. Studies in sensory axons branching and arborization have shown that Slit has a dual branch-repelling and branch-promoting functions, and that Slit could also function as a positive molecule to stimulate sensory axon branching and arborization [39]. Recently, Daseenco et al have identified Down syndrome cell adhesion molecule 1 (Dscam1) as the receptor to mediate the Slit positive function for sensory axon branching and arborisation. They found that Slit directly binds to Dscam1 and enhances Dscam1 interacting with the receptor tyrosine phosphatase 69D. This interaction stimulates Dscam1 dephosphorylation and confers axon branching [38].

The expression patterns of Slit1-3 and Robo1-2 have been most characterized in the developing rodent embryonic nervous system and the adult rat brain [5, 17–19], but a systematic study of their expression pattern in the adult rodent peripheral nervous system has not yet been performed. We are interested in the role of axon guidance molecules in adult peripheral nerve regeneration and we use the mouse sciatic nerve injury as a research model for our studies. In order to understand the function of Slit/Robo signaling in adult peripheral regeneration, our first approach was to fully map their expression in the adult peripheral nervous system. Here, we report a detailed expression of Slit1-3 and Robo1-2 in the intact mouse sciatic nerve as well as their expression in the nerve cell bodies localizing in the ventral spinal cord (motor neurons) and dorsal root ganglion (DRG, sensory neurons). Our results show that, in the adult mouse peripheral nervous system, Slit1-3 and Robo1-2 are expressed in the cell bodies and axons of both motor and sensory neurons. While Slit1 and Robo2 are only expressed in peripheral axons and their cell bodies alone, we also see expression of Slit2, Slit3 and Robo1 in the satellite cells of the DRG, Schwann cells and fibroblasts of peripheral nerves. Finally, we also demonstrate the expression of Robo1 in the blood vessels of peripheral nerves.

## Materials and methods

### Animals

All work involving animals was carried out according to Home Office regulation under the UK Animals (Scientific Procedures) Act 1986. Ethical approval for all experiments was granted by Plymouth University Animal Welfare and Ethical Review Board. Mice were housed in a 12 hour light/dark cycle with ad libitum access to food and water. Equal numbers of males and females were used for these experiments. Slit1 and Slit3 knockout mice used for antibody verification purposes have been previously described [40–41]. C57BL/6 mice were purchased from Charles River UK limited and GFAP-GFP (green fluorescent protein) mice were purchased from Jackson laboratory (Stock number: 003257). The GFAP-GFP mice express cytoplasmic GFP driven by the Glial fibrillary acidic protein gene promoter [42]. Homozygous PLP-GFP mice were obtained from Professor Thomas Misgeld (Technische Universität München, Germany) with permission from Professor Wendy Macklin (University of Colorado, USA). The PLP-GFP mice express cytoplasmic GFP driven by the mouse myelin proteolipid protein (PLP) gene promoter in both myelinating and non-myelinating Schwann cells [43].

### RT-PCR and qRT-PCR

Lumbar (L) 4-L5 spinal cord, L4 and L5 DRG and sciatic nerve samples were dissected out from two month old C57BL/6 mice. Total mRNA was extracted with miRNeasy Mini Kit (Qiagen, 217004) and first stand cDNA was synthesised with M-MLV reverse transcriptase (Promega, M368) using random hexanucleotide primers (Promega, C1181). Quantitative (q) PCR was performed in the PCR LightCycler480 Real-Time PCR Instrument (Roche Applied Science) using SYBR Green I Master mix. The following primers were used for both RT-PCR and qRT-PCR: Slit1 (forward: 5’-CTGCTCCCCGGATATGAACC-3’; reverse: 5’-TAGCATGCA CTCACACCTGG-3’), Slit2 (forward: 5’-AACTTGTACTGCGACTGCCA-3’; reverse: 5’-TCCTCATCACTGCAGACAAACT-3’), Slit3 (forward: 5’-AGTTGTCTGCCTTCCGACAG-3’; reverse: 5’-TTTCCATGGAGGGTCAGCAC-3’), Robo1 (forward: 5’-GCTGGCGACATGGGATCATA-3’; reverse: 5’-AATGTGGCGGCTCTTGAACT-3’); Robo2 (forward: 5’-CGAGCTCCTCCACAGTTTGT-3’; reverse: 5’-GTAGGTTCTGGCTGCCTTCT-3’), Robo3 (forward: 5’-CCTGTTCAAACCCAGGACAGC-3’; reverse: 5’-ACACACGGAATCCTTGCACC-3’), Robo4 (forward: 5’-AAGCCCAGGTCCAAACTCTG-3’; reverse: 5’-GTTGCGGTGAAGTTGTGGTC-3’); GAPDH (forward: 5’-AAGGTCATCCCAGAGCTGAA-3’; reverse: 5’-CTGCTTCACCACCTTCTTGA-3’). Cross point (Cp) values were calculated by using the software of the LightCycler480 Real-Time PCR Instrument. Relative Slit1-3 and Robo1-4 mRNA level were calculated by the -2^ΔΔC^T method [44] using GAPDH as a reference gene for normalization. All reactions were carried out in triplicate for statistical analysis. As a positive control for the Robo3 PCR primers, a plasmid containing mouse Robo3 (a gift from Professor Alain Chédotal, Institut de la Vision, Paris, France) was used [33].

### Primary and secondary antibodies for immunohistochemistry

The following primary antibodies were used for this study: Rabbit polyclonal to Slit1 (Sigma, SAB1307048, immunogen: synthetic peptide of N terminal human Slit1). Rabbit polyclonal to Slit2 (Chemicon, AB5701, immunogen: KLH-conjugated synthetic peptide corresponding to amino acids 1453–1528 of human Slit2). Rabbit polyclonal to Slit3 (Sigma, SAB2104337, immunogen: synthetic peptide of N terminal human Slit3, PRRLANKRISQIKSKKFRCSGSEDYRSRFSSECFMDLVCPEKCRCEGTIV). Rabbit polyclonal to Robo1 (Abcam, ab7279, immunogen: KLH conjugated synthetic peptide corresponding to amino acids 1632–1644 of human Robo1). Rabbit polyclonal to Robo2 (Santa Cruz, sc-25673, immunogen: amino acids 1281–1380 of human Robo2). NeuN (Merck Millipore, ABN91), Neurofilament heavy chain (Abcam, ab4680), CD31 (BD Pharmingen, 550274), Fibronectin (Santa Cruz, SC-6592).

Hoechst 33342 nuclear dye (H3570) and species specific secondary antibodies conjugated with Alexa Fluor 488 or 568 dyes were purchased from Thermo Scientific. The Hoechst dye (Ho) stain identifies cell nuclei within the tissue section.

### Immunohistochemistry

In total, 12 C57BL/6 adult animals were used to obtain L4-L5 spinal cord, L4 and L5 DRG and sciatic nerve samples for immunohistochemistry studies. L4-L5 spinal cord, L4 and L5 DRG and sciatic nerve samples were dissected out and fixed overnight in 4% paraformaldehyde/phosphate buffered saline (PBS) pH 7.4 (PFA) at 4°C. Samples were then washed in PBS (3x10 minutes) and dehydrated in 30% sucrose (in PBS) overnight at 4°C. Subsequently, samples were embedded into Optimal Cutting Temperature (OCT) medium and sectioned on a cryostat at 15μm thickness. For each tissue type, 4 sections from 4 different animals were randomly selected under light microscope for each antibody staining. 3 independent repeats have been stained for these studies. Tissue sections were permeabilised with 0.25% Triton X-100 plus 1% bovine serum albumin (BSA) in PBS for 30 minutes and then blocked with blocking buffer (3% BSA plus 0.05% Triton X-100 in PBS) for 45 minutes at room temperature. Subsequently, sections were incubated with primary antibodies (diluted in blocking buffer) overnight at 4°C. The following day, sections were washed with PBS (3x10 minutes) and then incubated with species specific secondary antibodies plus Hoechst dye (diluted in blocking buffer) for 1 hour at room temperature. Finally, sections were washed with PBS (3x10 minutes) and mounted with Citifluor (Agar Scientific, R1320) for imaging.

### In situ hybridization

Slit1-3 and Robo1-2 in situ probes were provided by Dr William Andrews (University College London, London, UK). L4 and L5 DRG were fixed in 4% PFA at 4°C overnight and then embedded in OCT for cryostat sections. 15μm thick sections were placed onto SuperFrost^®^Plus slides and then a Frame-Seal^™^ incubation chamber (Bio-rad, SLF1201) was added onto each slide. OCT was washed away with PBS and then 150μl of hybridisation buffer containing individual digoxigenin (DIG) labelled Slit1-3 or Robo1-2 probe was added into each incubation chamber. The incubation chambers were then sealed with cover slips. Slides were placed into a hybridisation box and kept at 65°C overnight for hybridisation. The following day, coverslips were removed from the incubation chambers and sections were washed 3 x 1 hour with a wash solution (50% formamide, 0.1% Tween-20, 150 mM NaCl and 15 mM sodium citrate) at 65°C. Sections were then washed 2 x 30 minutes with the MABT solution (100mM maleic acid, 150mM NaCl, 0.1% Tween-20, pH 7.5) at room temperature. Next, the sections were blocked with 500μl blocking solution (10% BSA in MABT solution) for one hour at room temperature. Anti-digoxigenin-alkaline phosphatase antibody (1:1500 in blocking solution) was applied to the chambers and incubated overnight at 4°C. Sections were washed 3 x 15 minutes with MABT solution at room temperature and signals were developed with nitroblue tetrazolium/5-bromo-4-chloro-3-indolyl phosphate solution (Roche Diagnostics GmbH; 1681451) at 37°C overnight. Slides were then washed 3x10 minutes in water and mounted for imaging on a Nikon fluorescence microscope (Eclipse 80i).

### Peripheral nerve surgery

Mice were anaesthetised with isoflurane, the right sciatic nerve was exposed and transected at approximately 0.5 cm distal to the sciatic notch. The muscle wound was closed with an 8.0 suture and the skin was closed with a surgical clip using an Autoclip applier (Fine Science Tools, 12020–09). All animals undergoing surgery were given appropriate post-operative analgesia (0.05% bupivacaine solution, topically applied above the muscle suture before applying surgical clip) and monitored daily. Four days after sciatic nerve transection, animals were euthanized humanely by cervical dislocation in accordance with UK Home Office regulations. Following this, the uncut control side sciatic nerve and the proximal and the distal nerve stumps of the transected nerve were then dissected out for western blotting analysis.

### Western blotting

Samples were directly lysed into a 1X SDS loading buffer. Proteins were separated on an 8% SDS polyacrylamide running gel and then transferred onto a polyvinylidene fluoride (PVDF, 0.45μm) transfer membrane using the wet transfer method. Membranes were blocked with 5% milk in TBST (Tris Buffered Saline plus 0.1% Tween-20) for one hour at room temperature. Slit1 (Sigma, SAB1307048) and Robo2 (Santa Cruz, sc-25673) primary antibodies were diluted (1:500 in TBST containing 5% milk) and the membranes were incubated in primary antibodies overnight at 4°C. The next day, the membranes were washed in TBST (3x10 minutes) and then incubated with HRP conjugated secondary antibody (1:5000 in 5% milk TBST) for one hour at room temperature. After three 10 minute TBST washes, Pierce ECL western blotting substrate was added onto the membrane and incubated for five minutes to develop the chemiluminescent signal. Amersham Hyperfilm^™^ ECL films were used to obtain the intensity of the chemiluminescent signal. Exposed films were then developed in a Compact X4 automatic film processor.

### Imaging

Immunostaining images were obtained using a Leica confocal microscope (TCS SP8). Several images were captured covering the entire field of interest. The individual images were then combined into one image using Adobe Photoshop software (Adobe Systems).

## Results

### Validation of Slit1-3 and Robo1-2 antibodies

**Fig 1:**
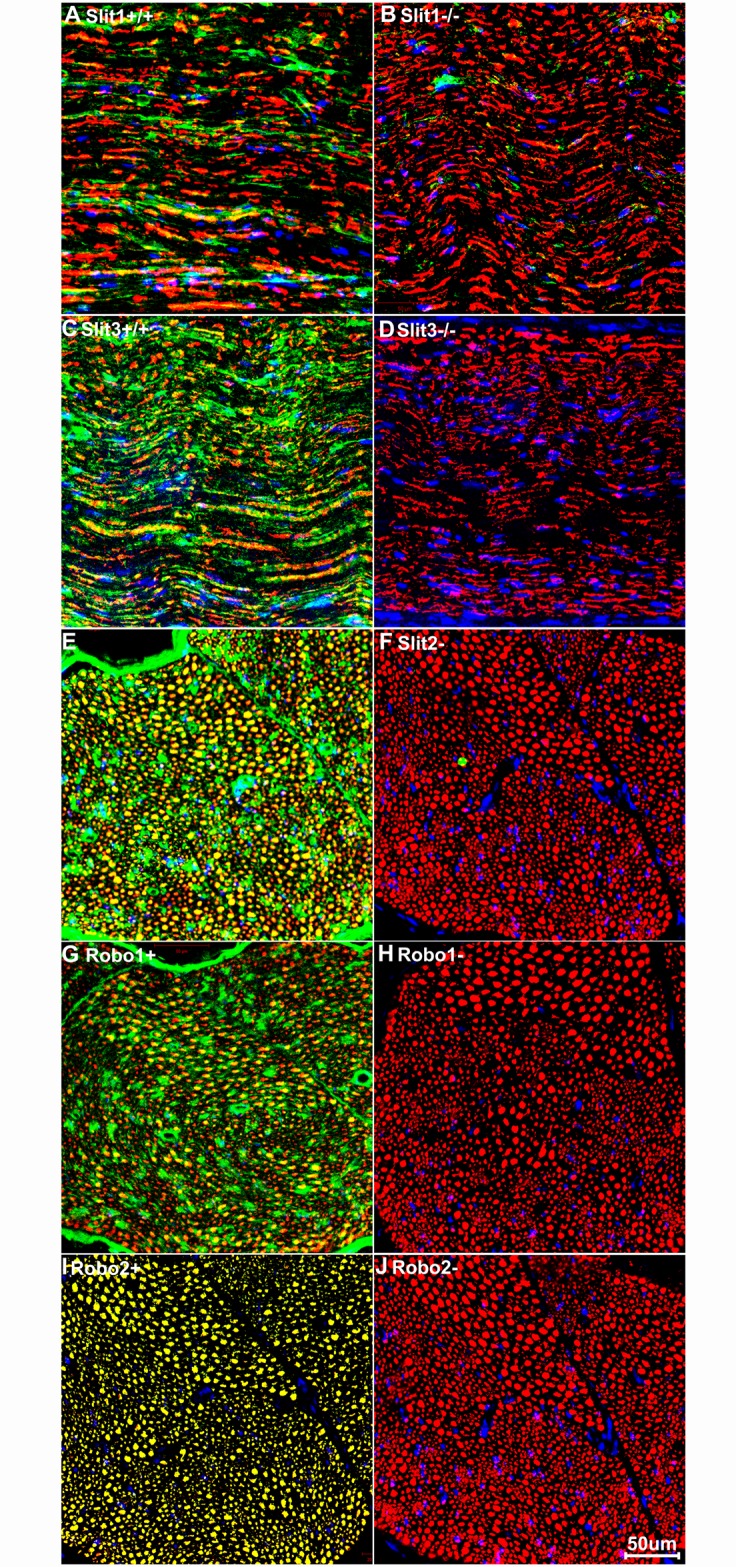
Validation of Slit1-3 and Robo1-2 antibodies. Staining with Slit1-3 and Robo1-2 antibodies is shown in green; staining with neurofilament heavy chain antibody is shown in in red. The yellow signal in panels A, C, E, G and I shows co-localization of the Slit1-3 and Robo1-2 signal with neurofilament staining. (A-B) Slit1 staining on transverse sections of sciatic nerve from Slit1 control (+/+, A) and Slit1 null (-/-, B) mice. (C-D) Slit3 staining on sciatic nerve samples from Slit3+/+ (C) and Slit3-/- (D) mice. (E-J) Slit2, Robo1 and Robo2 staining on sciatic nerve samples from C57BL/6 mice. Slit2, Robo1 and Robo2 primary antibodies were used in E, G and I but were omitted in F, H and J. Staining with Hoechst dye is also shown (blue) to identify cell nuclei within the tissue.

Before beginning the mapping of Slit/Robo expression in the adult mouse spinal cord and peripheral nervous system, we performed some preliminary analysis to verify the specificity of the Robo and Slit antibodies used in this study. Slit1-3 and Robo1-2 antibody selection were based upon previous publications: Slit1 [45], Slit2 [46–47], Robo1 [48–52] and Robo2 [53–54]. We are currently using Slit1-3 and Robo1-2 gene knockout mice [40–41, 55–56] in our work, and so these animals have been used, where possible, for antibody verification. The Slit1-/- mice are grossly normal [40] and 42% Slit3-/- mice survive longer than 5 months [41]. Therefore, we have validated the specificity of the Slit1 and Slit3 antibodies using sciatic nerve samples from two month old Slit1 null (-/-) and Slit3-/- mice. Slit1 antibody and Slit3 antibody show positive staining on sciatic nerve samples from Slit1 wild-type (+/+) and Slit3+/+ mice respectively (Fig 1A and 1C), but the staining pattern was not observed in sciatic nerve samples from Slit1-/- and Slit3-/- mice (Fig 1B and 1D). The Slit2-/-, Robo1-/- and Robo2-/- mouse pups die shortly after birth [40, 55–56], so we are unable to obtain adult sciatic nerve samples for staining with these antibodies. Therefore, we omitted Slit2, Robo1 and Robo2 primary antibodies to validate Slit2, Robo1 and Robo2 antibody staining using sciatic nerve samples from C57BL/6 mice. Staining signal is clearly visible with the Slit2, Robo1 and Robo2 antibodies (Fig 1E, 1G and 1I) but the signal is absent when the primary antibodies are omitted (Fig 1F, 1H and 1J). Therefore, we used these Slit1-3 and Robo1-2 antibodies for our immunohistochemistry studies.

### Measurement of the mRNA levels of Slit1-3 and Robo1-4 in adult spinal cord, dorsal root ganglion and sciatic nerve

**Fig 2:**
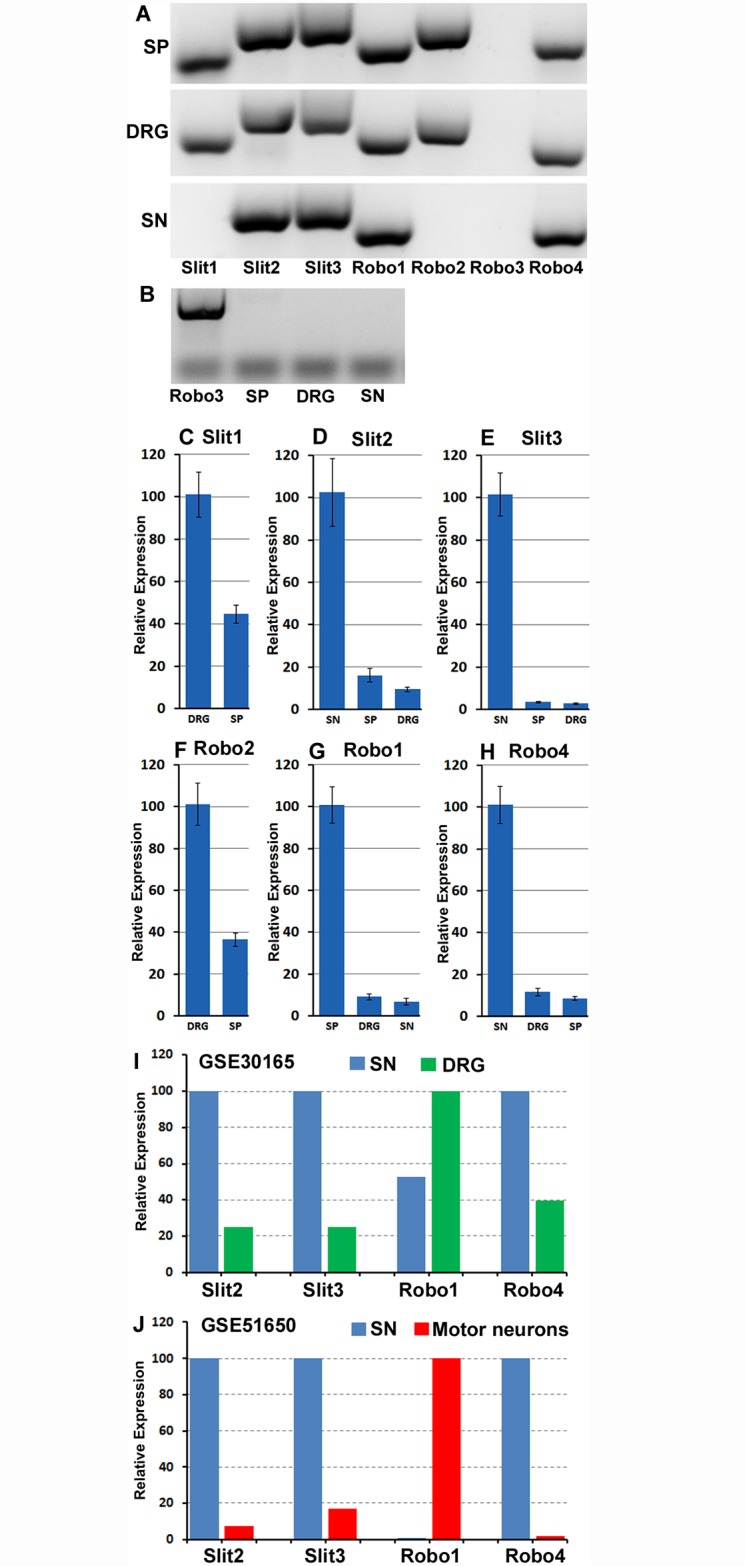
Slit1-3 and Robo1-4 mRNA expression in adult mouse (C57BL/6) spinal cord, DRG and sciatic nerve. (A) RT-PCR shows Slit1-3 and Robo1-4 mRNA expression in spinal cord (SP), DRG and sciatic nerve (SN). Slit1-3, Robo1-2 and Robo4 mRNA are expressed in spinal cord and DRG. In sciatic nerve, only Slit2, Slit3, Robo1 and Robo4 mRNA are detectable. Robo3 mRNA is not expressed in the adult mouse spinal cord and the peripheral nervous system. (B) Validation of Robo3 primers with a mouse Robo3 cDNA plasmid (Robo3). (C-H) qRT-PCR compares Slit1-3, Robo1-2 and Robo4 relative expression levels between spinal cord, DRG and sciatic nerve samples. The highest mRNA level has been set as 100% for each individual graph. Error bars show one standard deviation of the mean from 3 repeat experiments. Slit1 and Robo2 have their highest relative mRNA level in the DRG. Slit2, Slit3 and Robo4 have their highest mRNA level in the sciatic nerve. Robo1 has its highest mRNA level in spinal cord. (I-J), comparison of Slit2, Slit3, Robo1 and Robo4 relative levels from two microarray data sets (GSE30165 and GSE51650). As for Panels C-H, the highest mRNA level has been set as 100% for each individual graph. This analysis also shows that Slit2, Slit3 and Robo4 appear to have their highest mRNA level in the sciatic nerve. Robo1 appears to have its highest mRNA level in the motor neurons of the spinal cord.

The sciatic nerve contains two populations of axons, motor axons whose cell bodies localize in the ventral horn of spinal cord (L3-L6) and sensory axons, the cell bodies of which localize in the dorsal root ganglion (DRG) (L3-L6). As a first approach to analyse Slit and Robo expression, we used RT-PCR to analyse Slit1-3 and Robo1-4 expression in two month old C57BL/6 mouse sciatic nerve (SN), spinal cord (SP, L4-L5) and DRG (L4 and L5). Our RT-PCR results showed that Slit1-3, Robo1-2 and Robo4 mRNA are abundantly expressed in the adult mouse spinal cord and DRG but Robo3 mRNA is undetectable in both these tissues (Fig 2A). In the sciatic nerve, we could only detect Slit2, Slit3, Robo1 and Robo4 mRNAs. Slit1, Robo2 and Robo3 mRNA were all undetectable in the sciatic nerve (Fig 2A). We were also unable to detect Robo3 in spinal cord, DRG and sciatic nerve samples. Therefore, we used a mouse Robo3 cDNA plasmid as positive control to validate the Robo3 primers. Using mouse Robo3 cDNA as a positive control, we show that the Robo3 primers strongly amplifies Robo3 cDNA but Robo3 mRNA is not expressed in the adult mouse spinal cord, DRG or sciatic nerve (Fig 2B).

As revealed by our RT-PCR (Fig 2A and 2B), Robo3 is not expressed in the spinal cord, DRG or sciatic nerve. Next, we used quantitative (q) RT-PCR to compare the relative mRNA levels of Slit1-3, Robo1-2 and Robo4 between the spinal cord, DRG and sciatic nerve samples. qRT-PCR analysis was not performed for Slit1 and Robo2 in sciatic nerve as no expression was detected by RT-PCR in this tissue (Fig 2A). This quantitative analysis showed that Slit1 has its highest mRNA level in the DRG compared to the spinal cord (Fig 2C). Similar to Slit1, Robo2 also has its highest mRNA level in the DRG with less expression in the spinal cord (Fig 2F). Slit2, Slit3 and Robo4 show their highest expression in the sciatic nerve (Fig 2D, 2E and 2H), whereas Robo1 has it highest mRNA level in spinal cord (Fig 2G).

To further validate our qRT-PCR expression data, we also analysed published microarray data sets from other studies in the Gene Expression Omnibus (http://www.ncbi.nlm.nih.gov/geo/) that have studied gene expression signatures in adult mouse or rat sciatic nerve, DRG and motor neurons. In order to compare the level of Slit1-3 and Robo1-2 between sciatic nerve, DRG and motor neurons, we searched for microarray data sets that have analysed gene expression from at least two different tissue types of spinal cord, DRG or sciatic nerve in the same microarray. We found only two data sets (GSE30165 and GSE51650) matching this requirement. GSE30165 data set (http://www.ncbi.nlm.nih.gov/geo/query/acc.cgi?acc=GSE30165) measured gene expression in the adult rat (Sprague-Dawley, male, 180-220g) DRG and sciatic nerve in the same microarray [57]. The GSE51650 data set (http://www.ncbi.nlm.nih.gov/geo/query/acc.cgi?acc=GSE51650) measured gene expression in two month old C57BL/6 mouse motor neurons (isolated by laser capture microdissection) and sciatic nerve at the same time [58]. Both data sets have used sciatic nerve samples and we know from our analysis above that only Slit2, Slit3, Robo1 and Robo4 mRNAs are present in the sciatic nerve (Fig 2A). Therefore, we have compared the relative expression levels of Slit2, Slit3, Robo1 and Robo4 in both of these previously published data sets (Fig 2I and 2J). In agreement with our qRT-PCR data, analysis of both published microarray data sets also shows that Slit2, Slit3 and Robo4 have a higher relative expression in sciatic nerve as compared to DRG (GSE30165, Fig 2I) and motor neurons (GSE51650, Fig 2J). For Robo1 in the GSE51650 data set, it has its highest level in motor neurons (Fig 2J) as compared to sciatic nerve, correlating with the high relative expression we see in spinal cord as compared to DRG and sciatic nerve (Fig 2G).

Our qRT-PCR amplification curves in the sciatic nerve showed that Robo1 required the highest number of amplification cycles to reach the cross point, Robo4 was second and Slit2 third. In contrast, Slit3 requires the lowest amplification cycles to reach the cross point. Although the amplification efficiency may be different between Slit2, Slit3, Robo1 and Robo4 primers, the amplification cycle number would suggest that Slit3 has the highest relative mRNA level in the sciatic nerve, Slit2 is second highest and Robo4 is the third. Robo1 has the lowest mRNA level in the sciatic nerve. Correspondingly, both published microarray data sets showed that Slit3 has the highest expression value and Robo1 the lowest expression value in sciatic nerve (expression values not shown [57–58]).

From the above results, we know that Robo3 is not expressed in the adult mouse spinal cord and peripheral nervous system. In addition, Robo4 is known to be an endothelial cell specific protein and the evidence that the Slit1-3 ligands bind to Robo4 is currently very poor [25–30]. Therefore, in the following experiments, we focused on the mapping of Slit1-3 and Robo1-2 expression in the spinal cord, DRG and sciatic nerve.

### Sensory neurons in the DRG express Slit1-3 and Robo1-2

**Fig 3:**
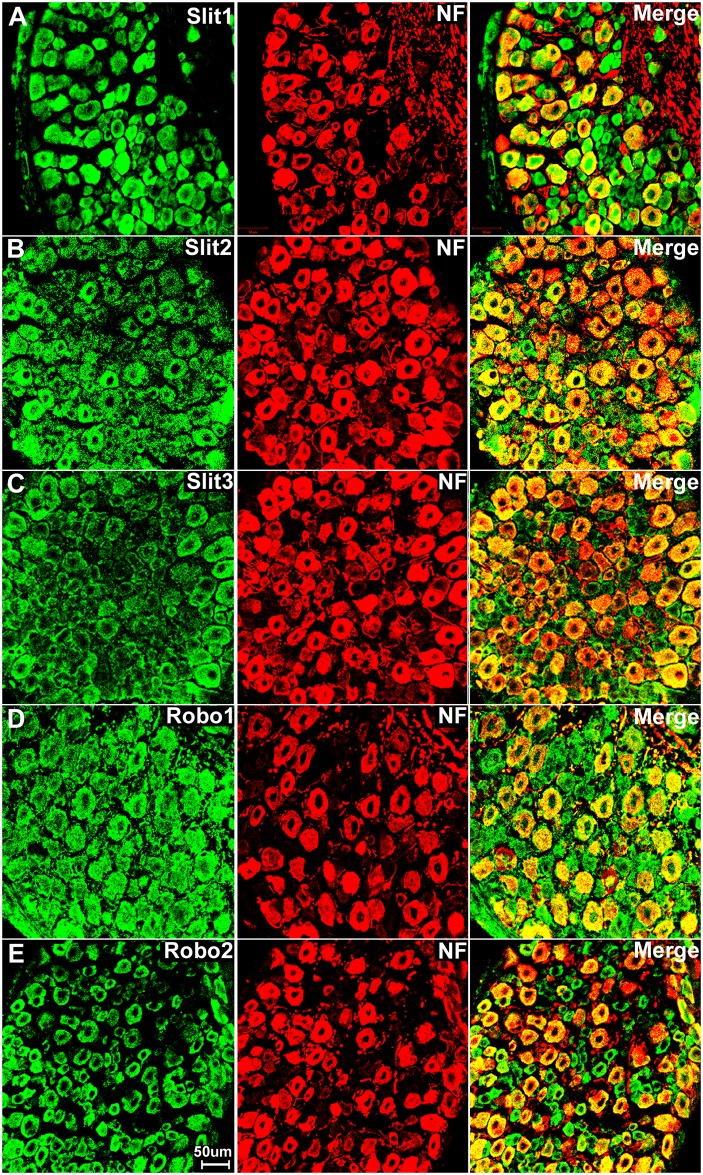
Double staining of Slit1-3 and Robo1-2 with neurofilament heavy chain in DRG. The neurofilament heavy chain antibody (NF) labels large diameter sensory neurons within the DRG. Merged images show that Slit1 (A), Slit2 (B), Slit3 (C), Robo1 (D) and Robo2 (E) are seemingly all expressed by large diameter sensory neurons. The yellow colour in merged images shows the co-localization of Slit1-3 and Robo1-2 signal with neurofilament heavy chain staining. Slit1-3 and Robo1-2 also appear to be expressed in small diameter cells in addition to their expression in large diameter sensory neurons. Slit1 (A) and Robo2 (E) show clear neuronal cell body staining. In contrast, Slit2 (B), Slit3 (C) and Robo1 (D) also show positive staining in the gaps between the cell bodies of sensory neurons in addition to their expression in the neuronal cell bodies.

**Fig 4:**
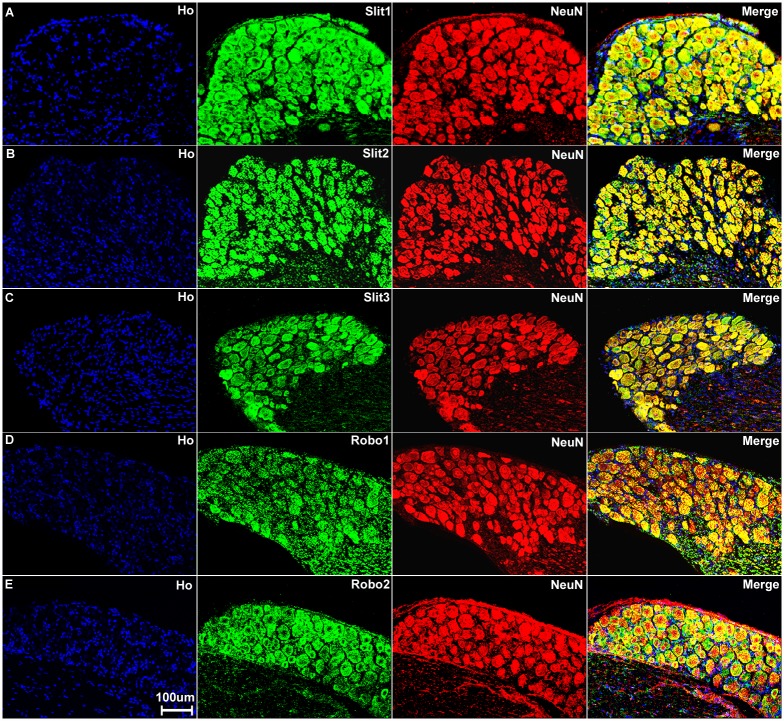
Sensory neurons in the DRG express Slit1-3 and Robo1-2. Double staining of Slit1-3 and Robo1-2 with NeuN in the DRG shows all sensory neurons in the DRG express Slit1-3 and Robo1-2. The neuronal marker NeuN labels both large and small diameter sensory neurons. Merged images show that Slit1 (A), Slit2 (B), Slit3 (C), Robo1 (D) and Robo2 (E) are all expressed by both large and small diameter sensory neurons. The yellow colour in merged images shows the co-localization of Slit1-3 and Robo1-2 signal with NeuN staining. Double staining of NeuN with Slit1-3 and Robo1-2 antibodies thus confirms that all the sensory neurons in the DRG express Slit1-3 and Robo1-2. Staining with Hoechst dye (Ho) is also shown (blue) to identify cell nuclei within the tissue.

**Fig 5:**
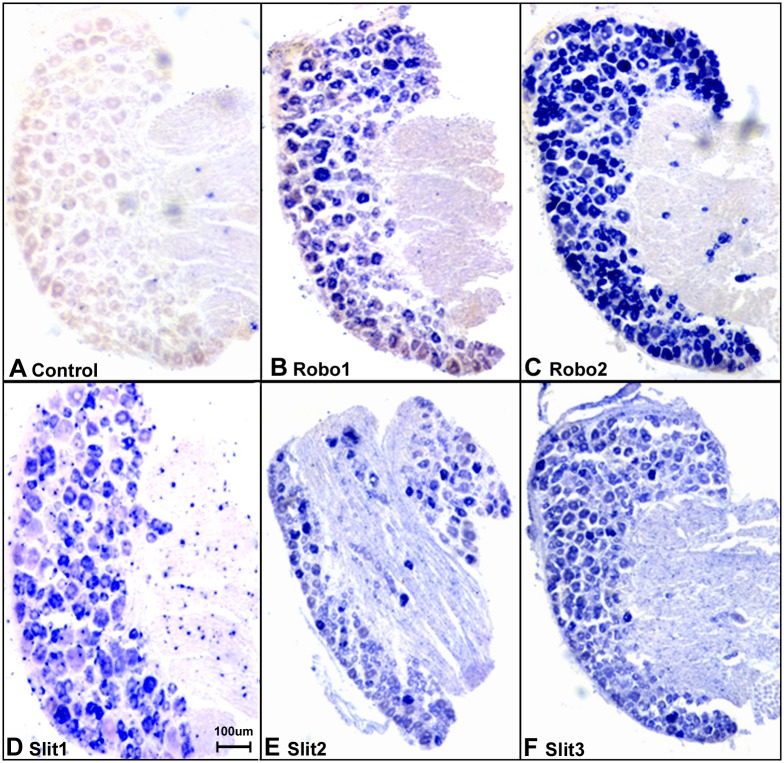
In situ hybridization confirms the expression of Slit1-3 and Robo1-2 in sensory neurons of the DRG. (A) Sense mRNA probe control, no blue signal has been developed in all the control samples using Slit1-3 and Robo1-2 sense probes for in situ hybridization (Robo1 sense mRNA probe control was shown in A, images for Robo2 and Slit1-3 sense mRNA probe control not shown). A positive signal for Robo1 (B), Robo2 (C), Slit1 (D), Slit2 (E) and Slit3 (F) were observed in sensory neuronal cell bodies. Slit2 (E) and Slit3 (F) signal also could be observed in the nerve in addition to their strong signal in the neuronal cell bodies of the DRG. The in situ hybridization results thus help to further confirm the specificity of the Slit1-3 and Robo1-2 antibodies used for immunohistochemistry.

Sensory neurons are part of the peripheral nervous system and they project an axon that has split into two branches, one branch runs to the periphery and the other into the spinal cord, while the cell bodies of the sensory neurons are within the DRG. First, we double stained DRG tissue sections with Slit1-3 or Robo1-2 antibodies together with the neuronal marker neurofilament heavy chain antibody to reveal Slit1-3 and Robo1-2 expression in the cell bodies of sensory neurons. The neurofilament heavy chain is a neural maker for large diameter sensory neurons in the DRG [59]. Double staining of neurofilament heavy chain with Slit1-3 and Robo1-2 antibodies showed that all the large diameter sensory neurons express Slit1-3 and Robo1-2 protein (Fig 3). Additional Slit1-3 and Robo1-2 staining also appeared to show expression of these proteins in other cell types within the DRG (see below). Next, we used the neuronal marker NeuN to label both large and small diameter sensory neurons. Double staining of NeuN with Slit1-3 and Robo1-2 antibodies showed that all sensory neurons in the DRG express Slit1-3 and Robo1-2 (Fig 4). To confirm this finding, we also performed in situ hybridization for Slit1-3 and Robo1-2 mRNAs in DRG tissue. In confirmation of the immunostaining results, the in situ hybridization shows that all the sensory neurons within the DRG appear to express Slit1-3 and Robo1-2 mRNAs (Fig 5).

**Fig 6:**
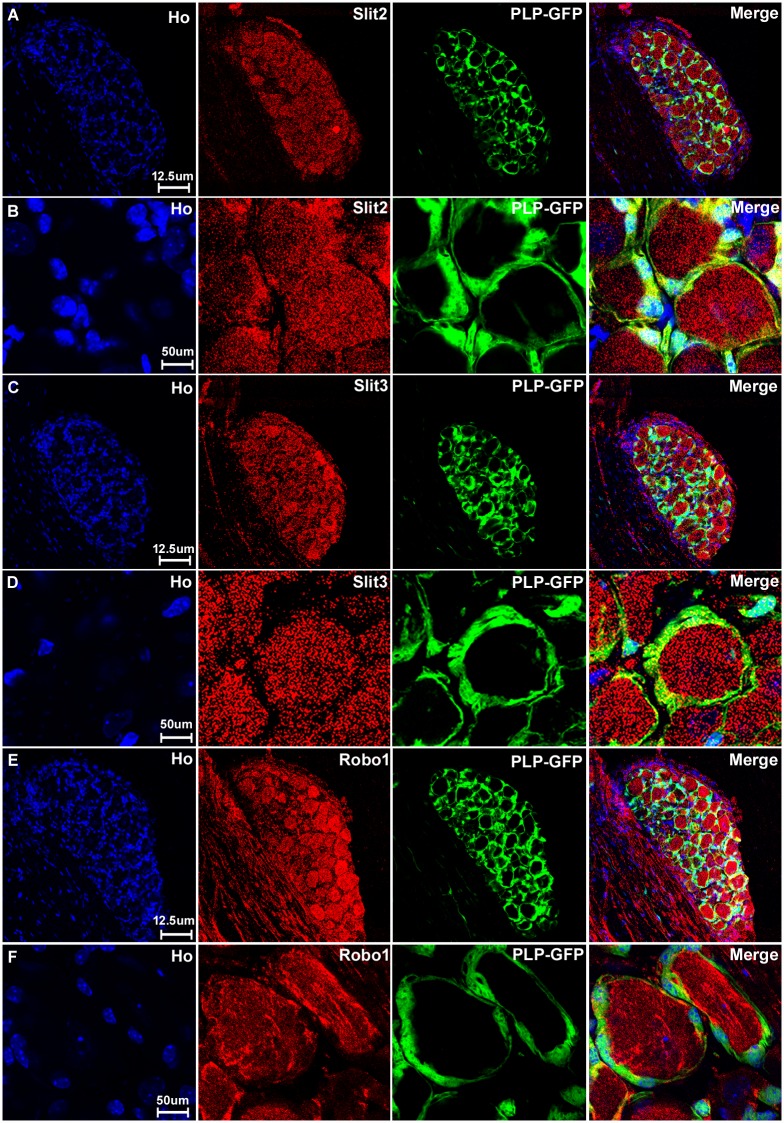
Satellite cells in the DRG express Slit2, Slit3 and Robo1. DRG tissues are from PLP-GFP transgenic mice which express cytoplasmic GFP within the satellite cells. Signals from Slit2-3 and Robo1 staining shows co-localization with GFP-positive satellite cells surrounding the neuronal cell bodies. Lower magnification images show Slit2 (A), Slit3 (C) and Robo1 (E) staining in the whole DRG sections. Higher magnification images show Slit2 (B), Slit3 (D) and Robo1 (F) signals co-localize with the GFP signal in satellite cells. Staining with Hoechst dye (Ho) is also shown (blue) to identify cell nuclei within the tissue.

With the staining of Slit1-3 and Robo1-2 in the DRG, we noticed that Slit1 and Robo2 show clear neuronal cell body staining only (Fig 3A and 3E). In contrast, Slit2, Slit3 and Robo1 also show positive staining between the cell bodies of the sensory neurons in the DRG (Fig 3B–3D). This indicates that cells surrounding the sensory neurons also express Slit2, Slit3 and Robo1. Satellite cells in the DRG are known to provide mechanical and metabolic support by surrounding the cell bodies of sensory neurons [60]. To test if these Slit2, Slit3 and Robo1 positive cells are indeed satellite cells, we stained the DRG tissue with Slit1-3 and Robo1-2 antibodies in PLP-GFP transgenic mice which express cytoplasmic green fluorescent protein GFP within the satellite cells of the DRG [43]. Staining within the DRG tissue of the PLP-GFP mice indicates that GFP-positive satellite cells in the DRG also express Slit2, Slit3 and Robo1 protein (Fig 6). Thus, consistent with our qRT-PCR results that Slit1-3 and Robo1-2 mRNA are all expressed in the DRG, our immunohistochemistry shows that all the sensory neurons in DRG express Slit1-3 and Robo1-2 proteins and that the satellite cells within the DRG also appear to express Slit2, Slit3 and Robo1 (Fig 6).

### Expression of Slit1-3 and Robo1-2 in adult mouse spinal cord

**Fig 7:**
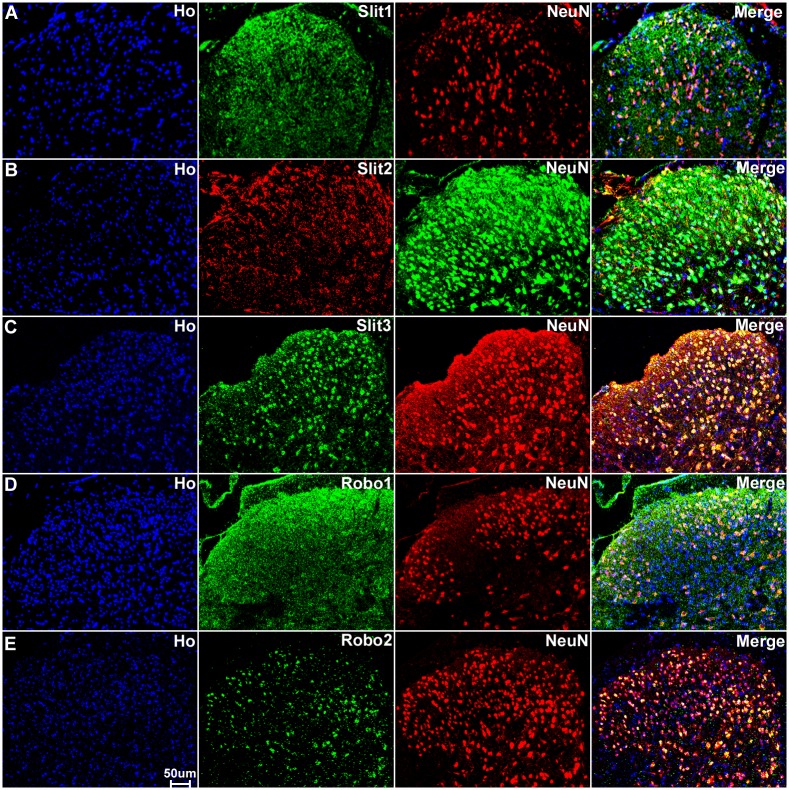
Slit1-3 and Robo1-2 expression in the dorsal region of adult mouse spinal cord. Slit1-3 and Robo1-2 double staining with NeuN to show Slit1-3 and Robo1-2 expression in the dorsal region of adult mouse spinal cord. Spinal cord sections are from the L4/L5 region of adult C57BL/6 mice. Double staining with NeuN showed that Slit1 (A) and Robo1 (D) are not only expressed in dorsal neuronal cell bodies but are also expressed in other cells in the grey matter of the dorsal spinal cord. Slit2 (B) expression is low in dorsal spinal cord and seemingly restricted to neuronal cell bodies. Slit3 (C) and Robo2 (E) are also mainly expressed in the neuronal cell bodies in the dorsal spinal cord. Staining with Hoechst dye (Ho) is also shown (blue) to identify cell nuclei within the tissue.

**Fig 8:**
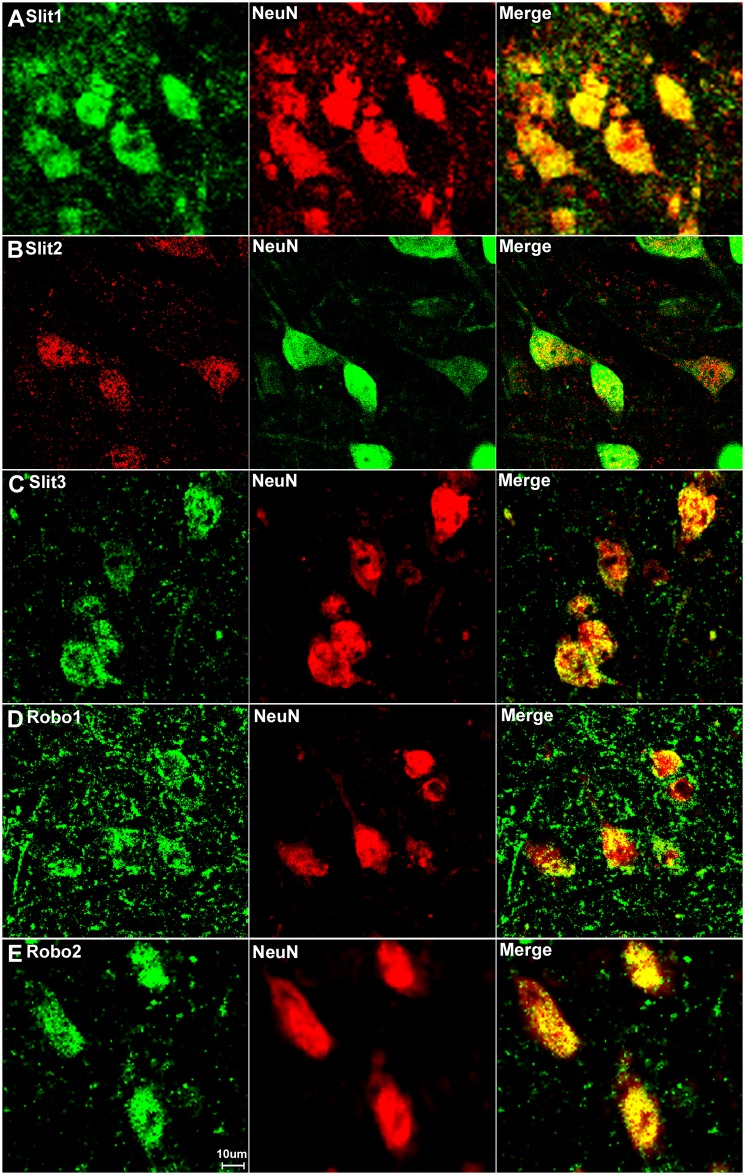
Slit1-3 and Robo1-2 expression in the ventral region of adult mouse spinal cord. Images show Slit1-3 and Robo1-2 expression in the neuronal cell bodies localizing in the ventral horn of the spinal cord. (A) Slit1, (B) Slit2, (C) Slit3, (D) Robo1 and (E) Robo2. Slit1-3 and Robo2 are mainly expressed in the neuronal cell bodies in the ventral horn of the spinal cord. Robo1 is highly expressed in the ventral horn neuronal cell bodies of the spinal cord but Robo1 also appears to show expression in other cells in the grey matter of the spinal cord (D). Staining with Hoechst dye (Ho) is also shown (blue) to identify cell nuclei within the tissue.

**Fig 9:**
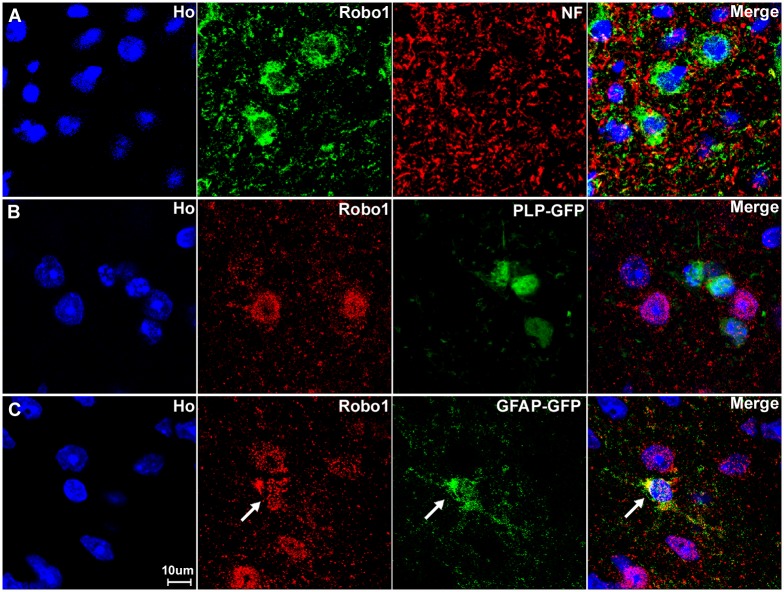
Astrocytes of the spinal cord express Robo1. (A) Double staining for Robo1 with neurofilament heavy chain antibody shows that very few axons within the spinal cord are Robo1 positive. (B) The Robo1 signal does not co-localize with the GFP signal in the spinal cord of PLP-GFP mice, which labels oligodendrocytes with cytoplasmic GFP. (C) The Robo1 signal co-localizes with the GFP signal in the spinal cord of GFAP-GFP mice, which labels astrocytes with GFP (indicated by white arrow). The staining confirms that Robo1 positive signal from outside of the neuronal cell bodies in the grey matter is from astrocytes. Staining with Hoechst dye (Ho) is also shown (blue) to identify cell nuclei within the tissue.

Our RT-PCR results have shown that Slit1-3 and Robo1-2 mRNAs are all present in the adult mouse spinal cord, but RT-PCR results do not reveal their expression pattern in different cell types of this tissue. To test whether Slit1-3 and Robo1-2 are expressed by the neurons in both the dorsal and the ventral horn of the spinal cord, we double stained NeuN with Slit1-3 and Robo1-2 antibodies on spinal cord sections from the L4/L5 region. In the dorsal spinal cord, Slit1 (Fig 7A) and Robo1 (Fig 7D) are not only expressed in neuronal cell bodies but also expressed in cells surrounding the neuronal cell bodies of the grey matter. Slit2 (Fig 7B) expression is very low in dorsal spinal cord. Slit3 (Fig 7C) and Robo2 (Fig 7E) are mainly expressed in the neuronal cell bodies in the dorsal spinal cord. In the ventral spinal cord, Slit1-3 and Robo2 are mainly expressed in the neuronal cell bodies (Fig 8A–8C and 8E). Similar to its expression pattern in the dorsal region, Robo1 is expressed in both neuronal cell bodies and cells surrounding the neuronal cell bodies of the grey matter in the ventral spinal cord (Fig 8D). To identify the Robo1 positive signal outside the neuronal cell bodies, we first double stained Robo1 with neurofilament heavy chain antibody to reveal Robo1 expression in axons of the grey matter. The staining showed that some axons are Robo1 positive (Fig 9) but the Robo1 positive signal is not completely co-localized with axons, indicating that other cell types in addition to neurons and axons in the grey matter also express Robo1. To identify non-neuronal Robo1 positive cells, we labelled with Robo1 antibodies on spinal cord tissue from both the GFAP-GFP and the PLP-GFP mice which express cytoplasmic GFP in astrocytes and oligodendrocytes respectively [42–43]. The staining showed that Robo1 co-localized with GFP in the spinal cord of GFAP-GFP mouse but not in the PLP-GFP mouse. Thus, in the grey matter of spinal cord, Robo1 is also expressed in the astrocytes of this region (Fig 9C).

Overall, our immunostaining on the spinal cord revealed that Slit1-3 and Robo1-2 are highly expressed in the neurons of the spinal cord including both dorsal and ventral neurons and that Robo1 is also expressed within astrocytes of the spinal cord.

### Slit1-3 and Robo1-2 expression in adult mouse sciatic nerve

**Fig 10:**
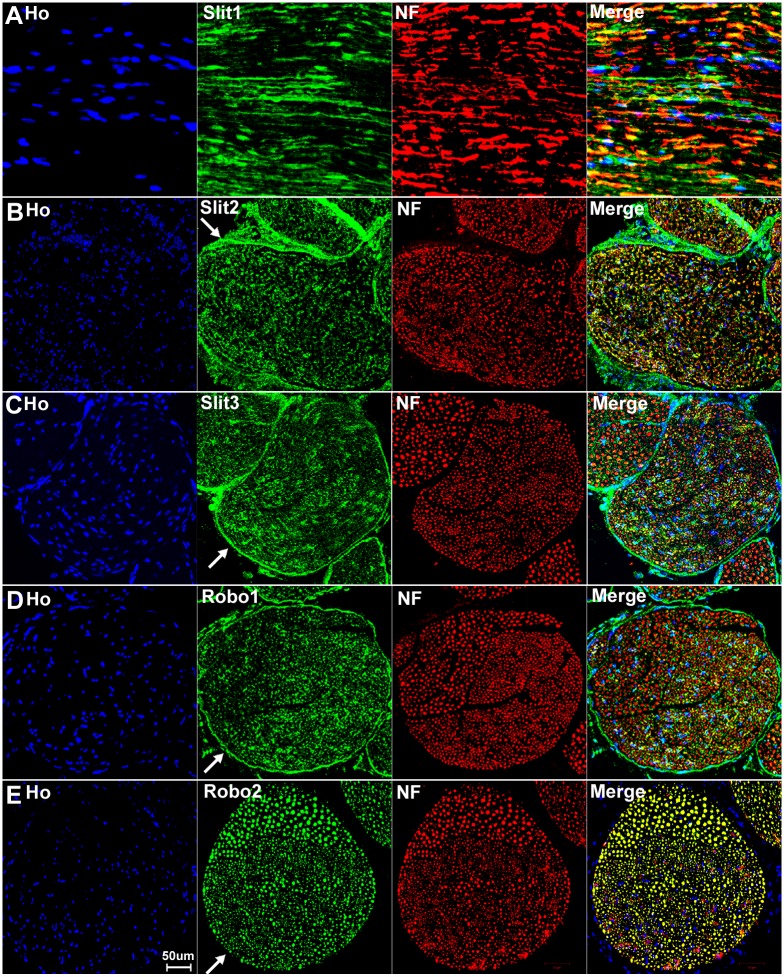
Expression of Slit1-3 and Robo1-2 in the adult mouse sciatic nerve. Lower magnification images show the expression of Slit1-3 and Robo1-2 in the adult mouse sciatic nerve by double staining of Slit1-3 and Robo1-2 antibody with the axon marker neurofilament heavy chain (NF). (A) Double staining of Slit1 with neurofilament heavy chain on a longitudinal sciatic nerve section shows that Slit1 is largely expressed in axons. Slit1 shows a better staining on sciatic nerve longitudinal sections compared to transverse sections. (B-D) Double staining of Slit2, Slit3 and Robo1 with neurofilament heavy chain on sciatic nerve transverse sections shows that Slit2, Slit3 and Robo1 are all expressed in axons. Slit2 (B), Slit3 (C) and Robo1 (D) also show positive staining outside the nerve fibres. In addition, the staining also shows that Slit2, Slit3 and Robo1 are expressed by cells in the nerve epineurium (indicated by white arrows). (E) Double staining of Robo2 with neurofilament heavy chain on sciatic nerve transverse section shows that the Robo2 signal completely co-localizes with the signal of the neurofilament heavy chain staining. Note that Robo2 is not expressed by the cells in the epineurium (indicated by white arrow in E). Staining with Hoechst dye (Ho) is also shown (blue) to identify cell nuclei within the tissue.

**Fig 11:**
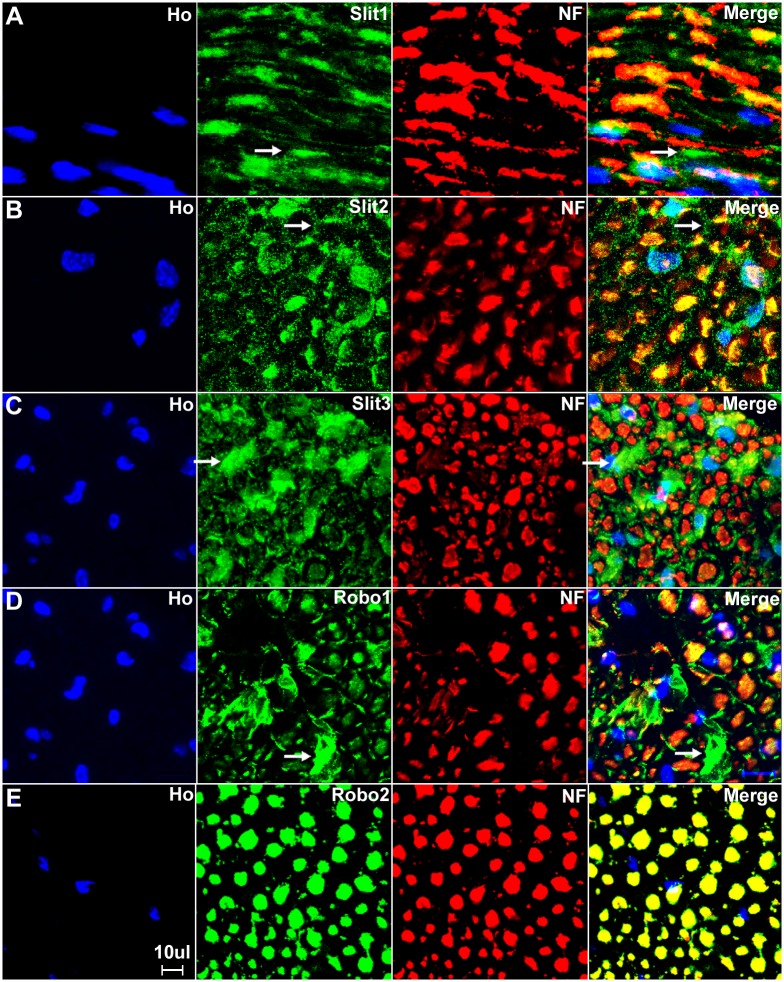
Higher magnification images from Fig 10 show Slit1-3 and Robo1-2 expression in the sciatic nerve. (A) Longitudinal sciatic nerve section staining with Slit1 antibody shows that Slit1 is largely expressed in axons, Slit1 staining could also be observed outside of axons (indicated by white arrow in A). The Slit1 positive staining outside the axons might be due to the secretion of Slit1 from axons. (B-D) Transverse sciatic nerve sections show Slit2 (B), Slit3 (C) and Robo1 (D) expression in axons and also positive staining in other cells other than axons (indicated by white arrows). (E) Robo2 staining shows complete co-localization of Robo2 with neurofilament heavy chain indicating that Robo2 is exclusively expressed in the axons of the peripheral nerves. Staining with Hoechst dye (Ho) is also shown (blue) to identify cell nuclei within the tissue.

**Fig 12:**
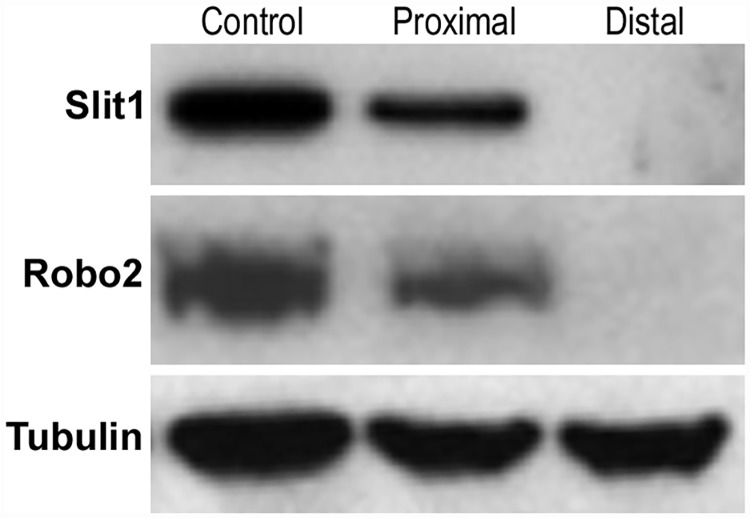
Slit1 and Robo2 protein are not expressed in the distal sciatic nerve stump after injury. Western blot shows the disappearance of Slit1 and Robo2 protein in the distal nerve stump following a sciatic nerve transection injury. Slit1 and Robo2 protein are present in the intact sciatic nerve (Control) and also within the proximal nerve stump 4 days after sciatic nerve transection injury. However, Slit1 and Robo2 protein completely disappeared in the distal nerve stump 4 days following a sciatic nerve transection injury. The disappearance of Slit1 and Robo2 protein in the distal nerve stump is not due to down-regulation because Slit1 and Robo2 mRNAs are undetectable in the intact adult sciatic nerve (see Fig 2A).

We have confirmed that the cell bodies of both motor and sensory neurons express Slit1-3 and Robo1-2. To test whether Slit1-3 and Robo1-2 protein are present in the axons of the peripheral nerve, we first double stained Slit1-3 and Robo1-2 antibody with the axon marker neurofilament heavy chain on longitudinal or transverse sections of sciatic nerve from adult C57BL/6 mice. This staining shows co-localization of Slit1-3 and Robo1-2 with neurofilament heavy chain indicating that Slit1-3 and Robo1-2 proteins are present within the axons of the peripheral nerves (Figs 10A–10E and 11A–11E). Slit1, Slit2 and Robo2 show strong expression in axons, whereas the expression of Slit3 and Robo1 is much weaker in axons compared to their expression in other cell types of the sciatic nerve (Figs 10A–10E and 11A–11E). Robo2 staining shows complete co-localization with neurofilament heavy chain indicating that Robo2 is exclusively expressed in the axons of the peripheral nerves (Figs 10E and 11E). In contrast, Slit1-3 and Robo1 show additional expression in other cell types of the sciatic nerve in addition to their expression within axons (Figs 10A–10D and 11A–11D). Both Slit1 and Robo2 mRNA and protein are expressed in the neuronal cell bodies of DRG and spinal cord (Figs 2–5, 7 and 8) and we could not detect expression of either Slit1 or Robo2 mRNAs in sciatic nerve by RT-PCR (Fig 2). For Slit1, this would suggest that the Slit1 protein is only expressed by neurons rather than other cell types in the sciatic nerve. The Slit1 positive staining outside the axons (Fig 11A) might be due to the secretion of Slit1 from axons. In order to confirm that the observed Slit1 protein originated from axons, we transected the sciatic nerve and used western blotting to study Slit1 expression in the distal nerve stump. The western blotting results clearly show that Slit1 protein is present in the intact sciatic nerve and the proximal nerve stump (Fig 12). However, Slit1 completely disappeared in the distal nerve stump due to the degradation and clearance of any axonal protein in the distal nerve stump 4 days after transection injury (Fig 12). We have also shown that Robo2 is an axonal protein by immunohistochemistry (Figs 10E and 11E). Western blot results for Robo2 show exactly the same pattern as Slit1 in the intact and injured sciatic nerve samples (Fig 12). Thus, for both Robo2 and Slit1, this suggests that they are only expressed by neurons and their associated axons within the peripheral nervous system.

**Fig 13:**
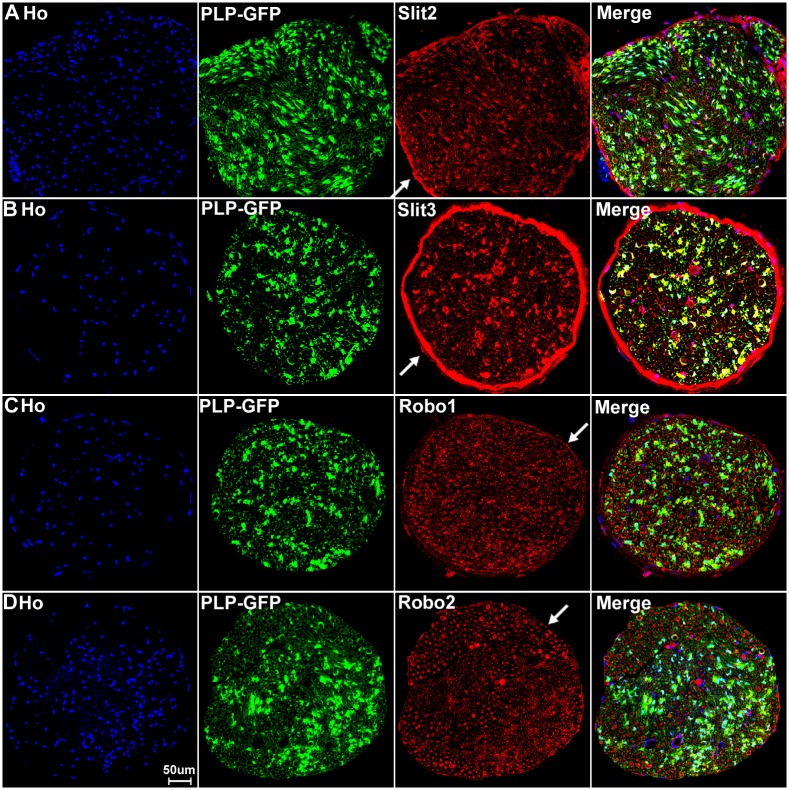
Slit2-3 and Robo1-2 staining on the sciatic nerve transverse sections from PLP-GFP mice. Lower magnification images show the staining of Slit2, Slit3, Robo1 and Robo2 on sciatic nerve transverse sections from PLP-GFP mice. The PLP-GFP mice express cytoplasmic GFP in both myelinating and non-myelinating Schwann cells. Slit2, Slit3 and Robo1 staining in the sciatic nerve transverse sections from PLP-GFP mice shows Slit2 (A), Slit3 (B) and Robo1 (C) co-localization with the GFP signal. In contrast, Robo2 (D) doesn’t show any co-localization with the GFP signal (D). Slit2, Slit3 and Robo1 are expressed by the cells in the epineurium (indicated by white arrows) but not Robo2 (D). Staining with Hoechst dye (Ho) is also shown (blue) to identify cell nuclei within the tissue.

**Fig 14:**
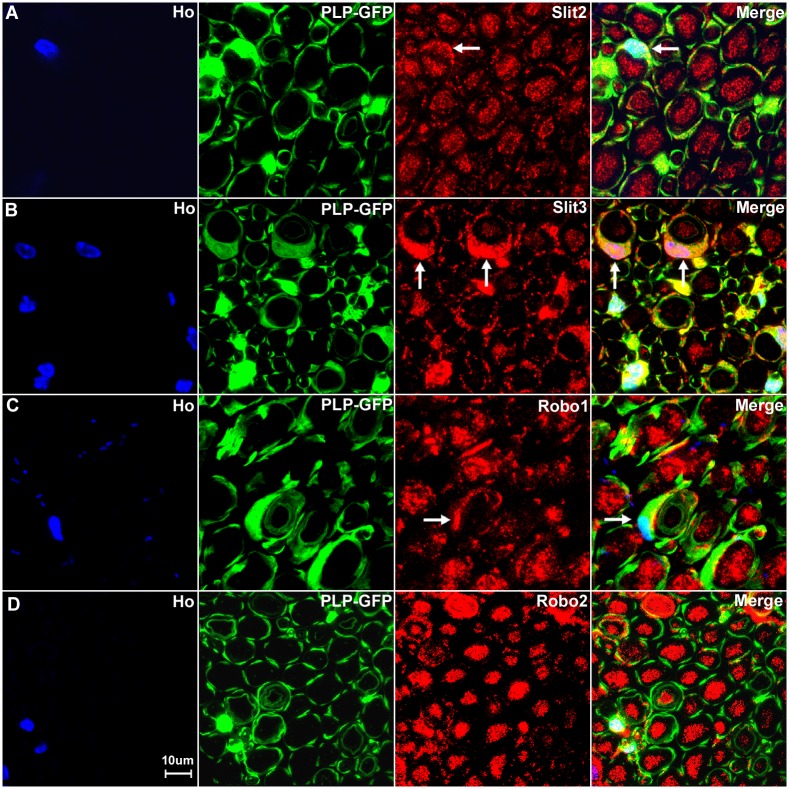
Slit2, Slit3 and Robo1 expression in the cell bodies of myelinating Schwann cells. Higher magnification images from Fig 13 show the expression of Slit2 (A), Slit3 (B) and Robo1 (C) in the cell bodies of myelinating Schwann cells (indicated by white arrows). Slit3 is highly expressed in the cell bodies of myelinating Schwann cells (B). In contrast, Robo2 staining does not show any co-localization with the GFP signal in further confirmation that Robo2 is expressed only in axons of the peripheral nerve (D). Staining with Hoechst dye (Ho) is also shown (blue) to identify cell nuclei within the tissue.

**Fig 15:**
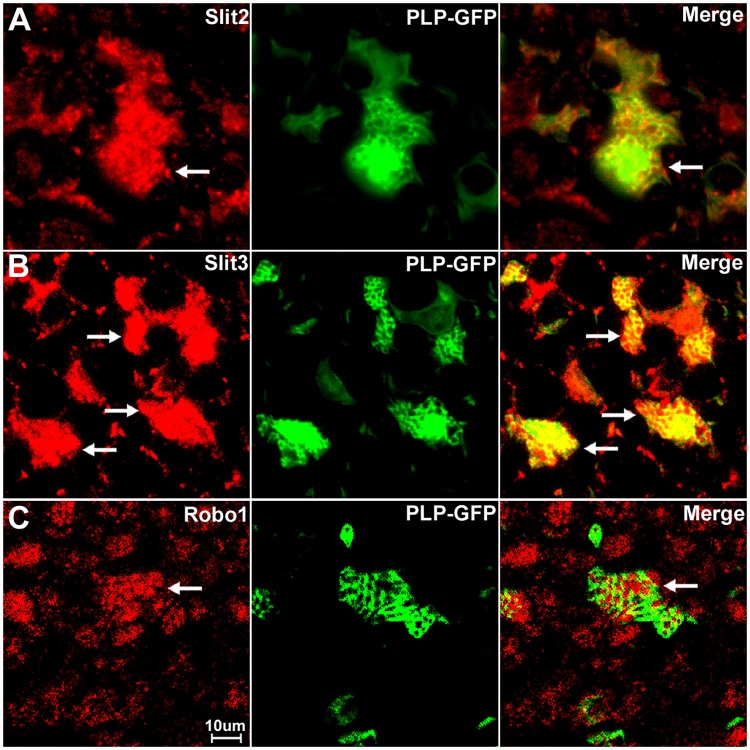
Slit2, Slit3 and Robo1 expression in the cell bodies of non-myelinating Schwann cells. Higher magnification images from Fig 13 show the expression of Slit2, Slit3 and Robo1 in non-myelinating Schwann cells. Non-myelinating Schwann cells in the PLP-GFP mouse nerve could be easily distinguished by the morphology of Remak bundles via the GFP signal (A-C). Slit2 (A), Slit3 (B) and Robo1 (C) expression in non-myelinating Schwann cells was observed (indicated by white arrows). Slit2 and Robo1 staining appeared stronger in the small diameter axons of the Remak bundle rather than the non-myelinating Schwann cells. Slit3 showed strong expression in non-myelinating Schwann cells.

Consistent with our RT-PCR results that Slit2, Slit3 and Robo1 mRNA are present in the sciatic nerve, our immunolabelling results show that Slit2, Slit3 and Robo1 also showed positive staining in other cell types in the sciatic nerve in addition to their expression in axons (Figs 10B–10D and 11B–11D). In order to identify these Slit2, Slit3 and Robo1 expressing cells, we first stained for Slit2, Slit3 and Robo1 on transverse sections of the sciatic nerve from the PLP-GFP mice which express GFP in the cytoplasm of Schwann cells within the nerve [43]. Immunolabelling with Slit2, Slit3 and Robo1 antibodies in the sciatic nerve of PLP-GFP mice demonstrated that myelinating Schwann cells expressed Slit2, Slit3 and Robo1 (Figs 13A–13C and 14). The strong staining of Slit3 in the cell body of myelinating Schwann cells suggests that Slit3 is highly expressed in the Schwann cells of the peripheral nerves (Fig 14B). A very weak overlap between Slit2 and Robo1 staining with PLP-GFP signal was also observed (Fig 14A and 14C). In contrast, Robo2 staining did not show any co-localization with the GFP signal, which further confirms that Robo2 is expressed only in axons (Figs 13D and 14D). During this study, we noticed that non-myelinating Schwann cells in the PLP-GFP mice could be easily distinguished by the morphology of Remak bundles via the GFP signal (Fig 15). Therefore, we were able to further show that Slit2, Slit3 and Robo1 are expressed in non-myelinating Schwann cells and their associated axons within the Remak bundles (Fig 15A–15C). The staining showed that Slit2 and Slit3 were also strongly expressed in non-myelinating Schwann cells (Fig 15A and 15B). Robo1 was also expressed in the non-myelinating Schwann cells but the Robo1 staining appeared stronger in the small diameter axons of the Remak bundle rather than the non-myelinating Schwann cells themselves (Fig 15C).

**Fig 16:**
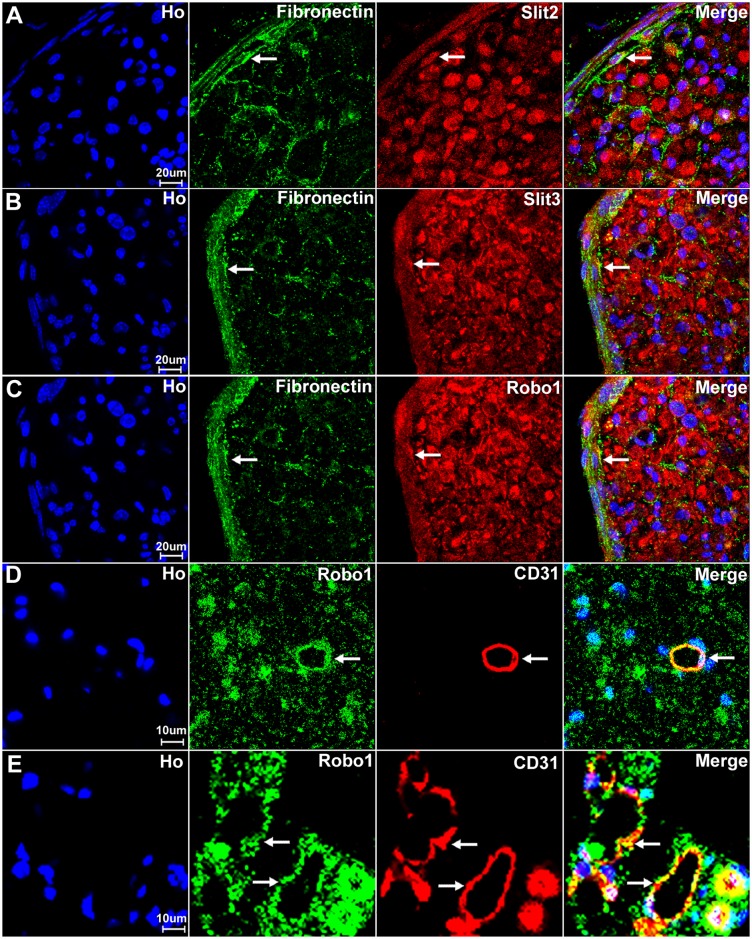
Slit2, Slit3 and Robo1 expression in fibroblasts and endothelial cells. Fibroblasts of the external nerve epineurium express Slit2, Slit3 and Robo1. Endothelial cells of the blood vessels in the sciatic nerve express Robo1. (A-C) Double staining of Slit2, Slit3 and Robo1 with fibronectin (a fibroblast marker) shows that epineurial fibroblasts also show expression of Slit2 (A), Slit3 (B) and Robo1 (C), indicated by white arrows. (D-E) Double staining of Slit2, Slit3 and Robo1 with CD31 (an endothelial cell marker) showing that blood vessels of the sciatic nerve express Robo1 (indicated by white arrows). (D) A CD31 positive blood vessel inside the sciatic nerve expresses Robo1. (E) Blood vessels outside the epineurium of the sciatic nerve also express Robo1. Staining with Hoechst dye (Ho) is also shown (blue) to identify cell nuclei within the tissue.

Our staining in both C57BL/6 and the PLP-GFP mice has shown that Slit2, Slit3 and Robo1 are also expressed by the cells in the external epineurium of the sciatic nerve (Figs 10B–10D and 13A–13C). The external epineurium is the outermost layer of connective tissue surrounding the peripheral nerve. Fibroblasts are known to be the major cell component of the external epineurium and they produce collagen fibres to form the backbone of the epineurium [61]. Therefore, we double stained Slit2, Slit3 and Robo1 with fibronectin (a fibroblast marker) on sciatic nerve sections and observed that epineurial fibroblasts express Slit2, Slit3 and Robo1 (Fig 16A–16C). We also stained Slit2, Slit3 and Robo1 with CD31 (an endothelial cell marker) to reveal their expression in blood vessels which supply the nerve. With the CD31 staining, we found that endothelial cells in the sciatic nerve express Robo1 (Fig 16D and 16E).

Thus, in the sciatic nerve, Slit1 and Robo2 are expressed by axons and Robo2 is exclusively localized to axons while Slit1 could be secreted by axons (Figs 11 and 12). Slit2, Slit3 and Robo1 are expressed by axons, together with myelinating and non-myelinating Schwann cells within the peripheral nerve. Fibroblasts of the epineurium also express Slit2, Slit3 and Robo1. Furthermore, Robo1 is expressed in the endothelial cells of the blood vessels in the sciatic nerve.

Finally, we summarize the expression pattern of Slit1-3 and Robo1-2 in adult mouse spinal cord, DRG and sciatic nerve in Table 1.

**Table 1:** Summary of Slit1-3 and Robo1-2 expression in adult mouse spinal cord and peripheral nervous system.

	L4-L5 spinal cord	L4 and L5 DRG	Sciatic nerve
**Slit1**	Dorsal neurons (++) Ventral neurons (++)	Sensory neurons (+++)	Axons (++)
**Slit2**	Dorsal neurons (+)Ventral neurons (++)	Sensory neurons (++)Satellite cells (++)	Axons (++)Myelinating Schwann cells (+++) Non-myelinating Schwann cells (+++)Fibroblasts (++)
**Slit3**	Dorsal neurons (++)Ventral neurons (++)	Sensory neurons (++)Satellite cells (++)	Axons (++)Myelinating Schwann cells (+++)Non-myelinating Schwann cells (+++)Fibroblasts (++)
**Robo1**	Dorsal neurons (++) Ventral neurons (+++)Astrocyte (+++)	Sensory neurons (++)Satellite cells (++)	Axons (++)Myelinating Schwann cells (++) Non-myelinating Schwann cells (++) Fibroblasts (++)Endothelial cells (++)
**Robo2**	Dorsal neurons (++)Ventral neurons (++)	Sensory neurons (+++)	Axons (++)
**Robo3**	Not expressed	Not expressed	Not expressed

^+:^ low expression;

^++:^ medium expression;

^+++:^ high expression

## Discussion

The expression patterns of Slit1-3 and Robo1-2 have been best characterized in the central nervous system during embryonic development [1–10, 19]. Slit2, Slit3 and Robo1-2 have been shown to be expressed by postnatal Schwann cells and Slit2 can act as repellent signal to regulate the migration of cultured rat Schwann cells [46]. In the adult central nervous system, Slit1-3 and Robo1-2 are differentially expressed at high levels in various regions of adult rat brain [5]. In the adult peripheral nervous system, Slit1 and Robo2 have been reported to be expressed in DRG neurons [55–56, 62–64]. However, a systematic study of the expression pattern of Slit1-3 and Robo1-2 in the adult peripheral nervous system has not yet been carried out. In this report, we have shown that Slit1-3 and Robo1-2 are differentially and topographically expressed in the adult mouse spinal cord and peripheral nervous system. Slit1-3 and Robo1-2 were all expressed by neurons of both the spinal cord and the DRG. In the intact sciatic nerve, Slit1 and Robo2 were expressed in axons and Slit1 may be secreted by axons. Slit2, Slit3 and Robo1 were expressed by axons, as well as myelinating and non-myelinating Schwann cells of the peripheral nerve. Fibroblasts of the epineurium also expressed Slit2, Slit3 and Robo1. Furthermore, Robo1 was also expressed in the endothelial cells of the blood vessels in the sciatic nerve.

Our qRT-PCR results showed that Slit2 and Slit3 have their highest mRNA expression in the sciatic nerve and we confirmed by immunohistochemistry that Slit2 and Slit3 were expressed by Schwann cells of the sciatic nerve. By immunohistochemistry, we also noticed that Slit2 and Slit3 were also highly expressed in the neurons of the spinal cord rather than in other cell types in the spinal cord. Considering the total mRNA for our qRT-PCR experiments is prepared from all the cell types of spinal cord, it is possible that neurons of the spinal cord express comparable levels of Slit2 and Slit3 mRNA to Schwann cells. However, the analysis of the GSE51650 microarray data set showed that the expression of Slit2 and Slit3 was higher in the sciatic nerve compared to the laser dissected motor neurons used in that study. Thus, we believe that Slit2 and Slit3 have their highest expression in the Schwann cell of the peripheral nervous system. Our qRT-PCR results showed that Slit3 required fewer amplification cycles to reach the cross point than Slit2 in the sciatic nerve samples which may imply a higher level of Slit3 mRNA than Slit2 mRNA in the sciatic nerve although the amplification efficiency might be different between Slit2 and Slit3 primers. Interestingly, both microarray data sets showed higher expression values for Slit3 than Slit2 in the sciatic nerve. Therefore, it is highly possible that the expression of Slit3 in the sciatic nerve is higher than that of Slit2.

Previously, Wang et al have reported that Schwann cells of the rat sciatic nerve express Slit2, Slit3, Robo1 and Robo2 [46]. Our results also showed that Schwann cells of the mouse sciatic nerve expressed Slit2, Slit3 and Robo1. However, we were unable to detect any Robo2 mRNA in the mouse sciatic nerve by RT-PCR. In contrast, Robo2 mRNA could be easily detected in both spinal cord and DRG. Our study indicates that Robo2 mRNA is not present in the Schwann cells of the peripheral nerve. We also showed that Robo2 protein was only present in the axons of the nerve but not in the Schwann cells by immunocytochemistry. Furthermore, our western blotting results (Fig 12) showed that Robo2 protein was undetectable in the distal nerve stump following sciatic nerve transection injury, confirming that Robo2 was not expressed by Schwann cells under these conditions. Therefore, we conclude that Robo2 is an axon specific protein in the peripheral nerves. In their paper, Wang et al showed that Slit2 could act as repellent to inhibit Schwann cell migration in culture and they suggested that this effect is mediated by both Robo1 and Robo2 receptors. Our results indicate this effect is likely mediated by only the Robo1 receptor in Schwann cells. Schwann cell migration plays a key role to guide axons across the nerve bridge after peripheral nerve transection injury [65–67]. It will be interesting to study how Slit-Robo1 signalling regulates Schwann cell migration into the nerve bridge during peripheral nerve regeneration.

We showed that Robo2 was exclusively expressed in axons of the sciatic nerve by immunocytochemistry and Robo2 mRNA was not expressed in the adult mouse sciatic nerve. Similar to Robo2, we were unable to detect any Slit1 mRNA in the mouse sciatic nerve by RT-PCR. In line with our result, Wang et al have also shown that Slit1 is not expressed by Schwann cells [51]. This indicates that Slit1 is only presented by axons rather than other cell types in the sciatic nerve. The Slit1 positive staining outside the axons might be due to the secretion of Slit1 from axons (Figs 10A and 11A). We further used Western blot analysis to show that Slit1 is absent from the distal nerve stump 4 days after sciatic nerve transection due to the degradation and clearance of any axonal protein in the distal nerve stump after injury (Fig 12). Thus, the same as Robo2, we conclude that Slit1 is expressed in axons and propose that the positive Slit1 staining outside axons is due to Slit1 secretion.

Robo3 expression has only been detected in the nervous system during embryonic development [21–24]. In the developing spinal cord and hindbrain, Robo3 is expressed at high levels on commissural axons before crossing and is gradually down-regulated after crossing [21–24]. Thus, it is not surprising that we couldn’t detect Robo3 expression in the adult peripheral nervous system. To date, there is no biological response associated with the direct binding of Slit ligands to Robo3 in mammals. A recent study has revealed that mammalian Robo3 does not bind Slit1-3 with high affinity due to the substitution of a few specific key residues in its Ig1 domain. Instead of binding to Slit1-3, mammalian Robo3 forms a protein complex with the Netrin-1 receptor DCC (Deleted in Colorectal Cancer) through their cytoplasmic domain [33]. Following Netrin-1 binding to DCC, a conserved tyrosine residue (Y1019) in the Robo3 cytoplasmic domain could be phosphorylated and this phosphorylation contributes to the attractive actions of Netrin-1 [33].

Using the zebrafish as a research model to study motor axon regeneration, Isaacman-Beck et al showed that regenerating motor axons select their original trajectory with high fidelity. They further showed that Schwann cells in the ventral projecting axons tract up-regulate col4a5 (Collagen type IV, alpha 5) after transection injury and col4a5 binds Slit1a to prevent dorsal projecting axons entering the ventral nerve track during regeneration [68]. They have reported that Slit1a is the only Slit1 isoform that is expressed by Schwann cells in the distal nerve of ventral motor axons after transection injury in zebrafish [68]. Our results show that motor and sensory axons of the peripheral nerve express both Robo1 and Robo2 receptors and that Schwann cells of the peripheral nerve express high levels of Slit2 and Slit3. It will be interesting to study the dynamic expression pattern of Slit1-3 and Robo1-2 in injured mouse peripheral nerves and their role in peripheral nerve regeneration. Our results of Slit1-3 and Robo1-2 expression patterns in the intact peripheral nerves could be used to compare how Slit1-3 and Robo1-2 change their expression pattern in response to peripheral nerve injury.

## References

[1] Kidd T, Bland KS, Goodman CS Slit is the midline repellent for the robo receptor in Drosophila. Cell 1999; 96: 785–794. 10102267 10.1016/s0092-8674(00)80589-9

[2] Wang KH, Brose K, Arnott D, Kidd T, Goodman CS, Henzel W, et al Biochemical purification of a mammalian slit protein as a positive regulator of sensory axon elongation and branching. Cell 1999; 96: 771–784. 10102266 10.1016/s0092-8674(00)80588-7

[3] Yuan W, Zhou L, Chen JH, Wu JY, Rao Y, Ornitz DM. The mouse SLIT family: secreted ligands for ROBO expressed in patterns that suggest a role in morphogenesis and axon guidance. Dev Biol. 1999; 212: 290–306. 10.1006/dbio.1999.9371 10433822

[4] Brose K, Tessier-Lavigne M. Slit proteins: key regulators of axon guidance, axonal branching, and cell migration. Curr Opin Neurobiol. 2000; 10: 95–102. 10679444 10.1016/s0959-4388(99)00066-5

[5] Marillat V, Cases O, Nguyen-Ba-Charvet KT, Tessier-Lavigne M, Sotelo C, Tessier-Lavigne M, et al Spatiotemporal expression patterns of slit and robo genes in the rat brain. J Comp Neurol. 2002; 442: 130–155. 11754167 10.1002/cne.10068

[6] Shu T, Sundaresan V, McCarthy MM, Richards LJ. Slit2 guides both precrossing and postcrossing callosal axons at the midline in vivo. J Neurosci. 2003; 23: 8176–8184. 12954881 10.1523/JNEUROSCI.23-22-08176.2003 PMC6740498

[7] Hammond R, Vivancos V, Naeem A, Chilton J, Mambetisaeva E, Andrews W, et al Slit-mediated repulsion is a key regulator of motor axon pathfinding in the hindbrain. Development 2005; 132: 4483–4495. 10.1242/dev.02038 16162649

[8] Andrews W, Barber M, Hernadez-Miranda LR, Xian J, Rakic S, Sundaresan V, et al The role of Slit-Robo signaling in the generation, migration and morphological differentiation of cortical interneurons. Dev Biol. 2008; 313: 648–658. 10.1016/j.ydbio.2007.10.052 18054781

[9] Blockus H, Chedotal A. Slit-Robo signaling. Development 2016; 143: 3037–3044. 10.1242/dev.132829 27578174

[10] Plump AS, Erskine L, Sabatier C, Brose K, Epstein CJ, Goodman CS, et al Slit1 and Slit2 cooperate to prevent premature midline crossing of retinal axons in the mouse visual system. Neuron 2002; 33: 219–232. 11804570 10.1016/s0896-6273(01)00586-4

[11] Yuan W, Rao Y, Babiuk RP, Greer JJ, Wu JY, Ornitz DM. A genetic model for a central (septum transversum) congenital diaphragmatic hernia in mice lacking Slit3. Proc Natl Acad Sci USA. 2003; 100: 5217–5222. 10.1073/pnas.0730709100 12702769 PMC154325

[12] Grieshammer U, Le M, Plump AS, Wang F, Tessier-Lavigne M, Martin GR. SLIT2-mediated ROBO2 signaling restricts kidney induction to a single site. Dev Cell 2004; 6: 709–717. 15130495 10.1016/s1534-5807(04)00108-x

[13] Anselmo MA, Dalvin S, Prodhan P, Komatsuzaki K, Aidlen JT, Schnitzer JJ, et al Slit and robo: expression patterns in lung development. Gene Expr Patterns 2003; 3: 13–19. 12609596 10.1016/s1567-133x(02)00095-9

[14] Mommersteeg MT, Andrews WD, Ypsilanti AR, Zelina P, Yeh ML, Norden J, et al Slit-roundabout signaling regulates the development of the cardiac systemic venous return and pericardium. Circ Res. 2013; 112: 465–475. 10.1161/CIRCRESAHA.112.277426 23255421

[15] Fujiwara M, Ghazizadeh M, Kawanami O. Potential role of the Slit/Robo signal pathway in angiogenesis. Vasc Med. 2006; 11: 115–121. 16886842 10.1191/1358863x06vm658ra

[16] Zhang B, Dietrich UM, Geng JG, Bicknell R, Esko JD, Wang L. Repulsive axon guidance molecule Slit3 is a novel angiogenic factor. Blood 2009; 114: 4300–4309. 10.1182/blood-2008-12-193326 19741192 PMC2774558

[17] Marillat V, Sabatier C, Failli V, Matsunaga E, Sotelo C, Tessier-Lavigne M, et al The slit receptor Rig-1/Robo3 controls midline crossing by hindbrain precerebellar neurons and axons. Neuron 2004; 69–79. 10.1016/j.neuron.2004.06.018 15233918

[18] Huminiecki L, Gorn M, Suchting S, Poulsom R, Bicknell R. Magic roundabout is a new member of the roundabout receptor family that is endothelial specific and expressed at sites of active angiogenesis. Genomics 2002; 79: 547–552. 10.1006/geno.2002.6745 11944987

[19] Sundaresan V, Mambetisaeva E, Andrews W, Annan A, Knoll B, Tear G, et al Dynamic expression patterns of Robo (Robo1 and Robo2) in the developing murine central nervous system. J Comp Neurol. 2004; 468: 467–481. 10.1002/cne.10984 14689480

[20] Qian L, Liu J, Bodmer R. Slit and Robo control cardiac cell polarity and morphogenesis. Curr Biol. 2005; 15: 2271–2278. 10.1016/j.cub.2005.10.037 16360689

[21] Sabatier C, Plump AS, Le M, Brose K, Tamada A, Murakami F, et al The divergent Robo family protein rig-1/Robo3 is a negative regulator of slit responsiveness required for midline crossing by commissural axons. Cell 2004; 117: 157–169. 15084255 10.1016/s0092-8674(04)00303-4

[22] Black DL, Zipursky SL. To cross or not to cross: alternatively spliced forms of the Robo3 receptor regulate discrete steps in axonal midline crossing. Neuron 2008; 58: 297–298. 10.1016/j.neuron.2008.04.019 18466738 PMC2703741

[23] Michalski N, Babai N, Renier N, Perkel DJ, Chedotal A, Schneggenburger R. Robo3-driven axon midline crossing conditions functional maturation of a large commissural synapse. Neuron 2013; 78: 855–868. 10.1016/j.neuron.2013.04.006 23664551

[24] Comer JD, Pan FC, Willet SG, Haldipur P, Millen KJ, Wright CV, et al Sensory and spinal inhibitory dorsal midline crossing is independent of Robo3. Front Neural Circuits 2015; 9: 36 10.3389/fncir.2015.00036 26257608 PMC4511845

[25] Suchting S, Heal P, Tahtis K, Stewart LM, Bicknell R. Soluble Robo4 receptor inhibits in vivo angiogenesis and endothelial cell migration. FASEB J. 2006; 19: 121–123. 10.1096/fj.04-1991fje 15486058

[26] Kaur S, Castellone MD, Bedell VM, Konar M, Gutkind JS, Ramchandran R. Robo4 signaling in endothelial cells implies attraction guidance mechanisms. J Biol Chem. 2006; 281: 11347–11356. 10.1074/jbc.M508853200 16481322

[27] Jones CA, London NR, Chen H, Park KW, Sauvaget D, Ramchandran R. Robo4 stabilizes the vascular network by inhibiting pathologic angiogenesis and endothelial hyperpermeability. Nat Med. 2008; 14: 448–453. 10.1038/nm1742 18345009 PMC2875252

[28] Jones CA, Nishiya N, London NR, Zhu W, Sorensen LK, Chan AC, et al Slit2-Robo4 signalling promotes vascular stability by blocking Arf6 activity. Nat Cell Biol. 2009; 11: 1325–1331. 10.1038/ncb1976 19855388 PMC2854659

[29] Marlow R, Binnewies M, Sorensen LK, Monica SD, Strickland P, Forsberg EC, et al Vascular Robo4 restricts proangiogenic VEGF signaling in breast. Proc Natl Acad Sci USA 2010; 107: 10520–10525. 10.1073/pnas.1001896107 20498081 PMC2890778

[30] Zhuang X, Cross D, Heath VL, Bicknell R. Shear stress, tip cells and regulators of endothelial migration. Biochem Soc Trans. 2011; 39: 1571–1575. 10.1042/BST20110746 22103489

[31] Evans TA, Santiago C, Arbeille E, Bashaw GJ. Robo2 acts in trans to inhibit Slit-Robo1 repulsion in pre-crossing commissural axons. Elife 2015; 4: e08407 10.7554/eLife.08407 26186094 PMC4505356

[32] Morlot C, Thielens NM, Ravelli RB, Hemrika W, Romijn RA, Gros P, et al Structural insights into the Slit-Robo complex. Proc Natl Acad Sci USA 2007; 104: 14923–14928. 10.1073/pnas.0705310104 17848514 PMC1975871

[33] Zelina P, Blockus H, Zagar Y, Peres A, Friocourt F, Wu Z, et al Signaling switch of the axon guidance receptor Robo3 during vertebrate evolution. Neuron 2014; 84: 1258–1272. 10.1016/j.neuron.2014.11.004 25433640

[34] Koch AW, Mathivet T, Larrivee B, Tong RK, Kowalski J, Pibouin-Fragner L, et al Robo4 maintains vessel integrity and inhibits angiogenesis by interacting with UNC5B. Dev Cell 2011; 20: 33–46. 10.1016/j.devcel.2010.12.001 21238923

[35] Smith-Berdan S, Nguyen A, Hassanein D, Zimmer M, Ugarte F, Ciriza J, et al Robo4 cooperates with CXCR4 to specify hematopoietic stem cell localization to bone marrow niches. Cell Stem Cell 2011; 8: 72–83. 10.1016/j.stem.2010.11.030 21211783 PMC3625377

[36] Smith-Berdan S, Nguyen A, Hong MA, Forsberg EC. ROBO4-mediated vascular integrity regulates the directionality of hematopoietic stem cell trafficking. Stem Cell Reports 2015; 4: 255–268. 10.1016/j.stemcr.2014.12.013 25640759 PMC4325232

[37] Wayburn B, Volk T. LRT, a tendon-specific leucine-rich repeat protein, promotes muscle-tendon targeting through its interaction with Robo. Development 2009; 136: 3607–3615. 10.1242/dev.040329 19793885

[38] Dascenco D, Erfurth ML, Izadifar A, Song M, Sachse S, Bortnick R, et al Slit and Receptor Tyrosine Phosphatase 69D Confer Spatial Specificity to Axon Branching via Dscam1. Cell 2015; 162: 1140–1154. 10.1016/j.cell.2015.08.003 26317474 PMC4699798

[39] Ma L, Tessier-Lavigne M. Dual branch-promoting and branch-repelling actions of Slit/Robo signaling on peripheral and central branches of developing sensory axons. J Neurosci 2007; 27: 6843–6851. 10.1523/JNEUROSCI.1479-07.2007 17581972 PMC6672698

[40] Plump AS, Erskine L, Sabatier C, Brose K, Epstein CJ, Goodman CS, et al Slit1 and Slit2 cooperate to prevent premature midline crossing of retinal axons in the mouse visual system. Neuron 2002; 33: 219–232. 11804570 10.1016/s0896-6273(01)00586-4

[41] Yuan W, Rao Y, Babiuk RP, Greer JJ, Wu JY, Ornitz DM. A genetic model for a central (septum transversum) congenital diaphragmatic hernia in mice lacking Slit3. Proc Natl Acad Sci 2003; 100: 5217–5222. 10.1073/pnas.0730709100 12702769 PMC154325

[42] Zhuo L, Sun B, Zhang CL, Fine A, Chiu SY, Messing A. Live astrocytes visualized by green fluorescent protein in transgenic mice. Dev Biol. 1997; 187: 36–42. 10.1006/dbio.1997.8601 9224672

[43] Mallon BS, Shick HE, Kidd GJ, Macklin WB. Proteolipid promoter activity distinguishes two populations of NG2-positive cells throughout neonatal cortical development. J Neurosci. 2002; 22: 876–885. 11826117 10.1523/JNEUROSCI.22-03-00876.2002 PMC6758537

[44] Livak KJ, Schmittgen TD. Analysis of relative gene expression data using real-time quantitative PCR and the 2(-Delta Delta C (T)) Method. Methods 2001; 25: 402–408. 10.1006/meth.2001.1262 11846609

[45] Yang YH, Manning Fox JE, Zhang KL, MacDonald PE, Johnson JD. Intraislet SLIT-ROBO signaling is required for beta-cell survival and potentiates insulin secretion. Proc Natl Acad Sci 2013; 110: 16480–16485. 10.1073/pnas.1214312110 24065825 PMC3799350

[46] Wang Y, Teng HL, Huang ZH. Repulsive migration of Schwann cells induced by Slit-2 through Ca2+-dependent RhoA-myosin signaling. Glia 2013; 61: 710–723. 10.1002/glia.22464 23361995

[47] Huang ZH, Wang Y, Su ZD, Geng JG, Chen YZ, Yuan XB, et al Slit-2 repels the migration of olfactory ensheathing cells by triggering Ca2+-dependent cofilin activation and RhoA inhibition. J Cell Sci 2011; 124: 186–197. 10.1242/jcs.071357 21187345 PMC3705990

[48] Guo SW, Zheng Y, Lu Y, Liu X, Geng JG. Slit2 overexpression results in increased microvessel density and lesion size in mice with induced endometriosis. Reprod Sci 2013; 20: 285–298. 10.1177/1933719112452940 22875847 PMC4077380

[49] Alajez NM, Lenarduzzi M, Ito E, Hui AB, Shi W, Bruce J, et al MiR-218 suppresses nasopharyngeal cancer progression through downregulation of survivin and the SLIT2-ROBO1 pathway. Cancer Res 2011; 71: 2381–2391. 10.1158/0008-5472.CAN-10-2754 21385904

[50] Ip BK, Bayatti N, Howard NJ, Lindsay S, Clowry GJ. The corticofugal neuron-associated genes ROBO1, SRGAP1, and CTIP2 exhibit an anterior to posterior gradient of expression in early fetal human neocortex development. Cereb Cortex 2011; 21: 1395–1407. 10.1093/cercor/bhq219 21060114 PMC3097990

[51] Sheldon H, Andre M, Legg JA, Heal P, Herbert JM, Sainson R, et al Active involvement of Robo1 and Robo4 in filopodia formation and endothelial cell motility mediated via WASP and other actin nucleation-promoting factors. FASEB J 2009; 23: 513–522. 10.1096/fj.07-098269 18948384 PMC4048916

[52] Fish JE, Wythe JD, Xiao T, Bruneau BG, Stainier DY, Srivastava D, et al A Slit/miR-218/Robo regulatory loop is required during heart tube formation in zebrafish. Development 2011; 138: 1409–1419. 10.1242/dev.060046 21385766 PMC3050667

[53] Yi XN, Zheng LF, Zhang JW, Zhang LZ, Xu YZ, Luo G, et al Dynamic changes in Robo2 and Slit1 expression in adult rat dorsal root ganglion and sciatic nerve after peripheral and central axonal injury. Neurosci Res. 2 56: 314–321. 10.1016/j.neures.2006.07.014 16979769

[54] Zhang HY, Zheng LF, Yi XN, Chen ZB, He ZP, Zhao D, et al Slit1 promotes regenerative neurite outgrowth of adult dorsal root ganglion neurons in vitro via binding to the Robo receptor. J Chem Neuroanat. 2011; 39: 256–261. 10.1016/j.jchemneu.2010.02.001 20172023

[55] Andrews W, Liapi A, Plachez C, Camurri L, Zhang J, Mori S, et al Robo1 regulates the development of major axon tracts and interneuron migration in the forebrain. Development 2006; 133: 2243–2252. 10.1242/dev.02379 16690755

[56] Grieshammer U, Le M, Plump AS, Wang F, Tessier-Lavigne M, Martin GR. SLIT2-mediated ROBO2 signaling restricts kidney induction to a single site. Dev Cell 2004; 6: 709–717. 15130495 10.1016/s1534-5807(04)00108-x

[57] Zhang Z, Yu B, Gu Y, Zhou S, Qian T, Wang Y, et al Fibroblast-derived tenascin-C promotes Schwann cell migration through beta1-integrin dependent pathway during peripheral nerve regeneration. Glia 2016; 64: 374–385. 10.1002/glia.22934 26497118

[58] Niemann A, Huber N, Wagner KM, Somandin C, Horn M, Lebrun-Julien F, et al The Gdap1 knockout mouse mechanistically links redox control to Charcot-Marie-Tooth disease. Brain 2014; 137: 668–682. 10.1093/brain/awt371 24480485 PMC3927703

[59] Ackerley S, Thornhill P, Grierson AJ, Brownlees J, Anderton BH, Leigh PN, et al Neurofilament heavy chain side arm phosphorylation regulates axonal transport of neurofilaments. J Cell Biol. 2003; 161: 489–495. 10.1083/jcb.200303138 12743103 PMC2172950

[60] Nascimento RS, Santiago MF, Marques SA, Allodi S, Martinez AM. Diversity among satellite glial cells in dorsal root ganglia of the rat. Braz J Med Biol Res. 2008; 41: 1011–1017. 19030716 10.1590/s0100-879x2008005000051

[61] Pina-Oviedo S, Ortiz-Hidalgo C. The normal and neoplastic perineurium: a review. Adv Anat Pathol 2008; 15: 147–164. 10.1097/PAP.0b013e31816f8519 18434767

[62] Piper M, Nurcombe V, Reid K, Bartlett P, Little M. N-terminal Slit2 promotes survival and neurite extension in cultured peripheral neurons. Neuroreport 2002; 13: 2375–2378. 12488830 10.1097/00001756-200212030-00041

[63] Bloechlinger S, Karchewski LA, Woolf CJ. Dynamic changes in glypican-1 expression in dorsal root ganglion neurons after peripheral and central axonal injury. Eur J Neurosci. 2004; 19: 1119–1132. 10.1111/j.1460-9568.2004.03262.x 15016071

[64] Chen ZB, Zhang HY, Zhao JH, Zhao W, Zhao D, Zheng LF, et al Slit-Robo GTPase-activating proteins are differentially expressed in murine dorsal root ganglia: modulation by peripheral nerve injury. Anat Rec (Hoboken) 2012; 295: 652–660. 22271578 10.1002/ar.22419

[65] Heermann S, Schwab MH. Molecular control of Schwann cell migration along peripheral axons: keep moving! Cell Adh Migr. 2013; 7: 18–22. 10.4161/cam.22123 23076214 PMC3544780

[66] Cattin AL, Burden JJ, Van Emmenis L, Mackenzie FE, Hoving JJ, Garcia Calavia N, et al Macrophage-Induced Blood Vessels Guide Schwann Cell-Mediated Regeneration of Peripheral Nerves. Cell 2015; 162: 1127–1139. 10.1016/j.cell.2015.07.021 26279190 PMC4553238

[67] Torigoe K, Tanaka HF, Takahashi A, Awaya A, Hashimoto K. Basic behavior of migratory Schwann cells in peripheral nerve regeneration. Exp Neurol 1996; 137: 301–308. 10.1006/exnr.1996.0030 8635545

[68] Isaacman-Beck J, Schneider V, Franzini-Armstrong C, Granato M. The lh3 Glycosyltransferase Directs Target-Selective Peripheral Nerve Regeneration. Neuron 2015; 88: 691–703. 10.1016/j.neuron.2015.10.004 26549330 PMC4655140

